# Coexisting gases regulate the rates of water adsorption by a flexible one-dimensional coordination polymer

**DOI:** 10.1039/d5sc01699a

**Published:** 2025-08-27

**Authors:** AnQi Wang, Xin Zheng, Yuki Saito, Arata Tateishi, Yuan Huang, Yuichi Kamiya, Hiroyasu Sato, Atsushi Kondo, Kiyonori Takahashi, Takayoshi Nakamura, Shin-ichiro Noro

**Affiliations:** a Graduate School of Environmental Science, Hokkaido University Sapporo 060-0810 Japan noro@ees.hokudai.ac.jp; b Faculty of Environmental Earth Science, Hokkaido University Sapporo 060-0810 Japan; c Rigaku Corporation Akishima 196-8666 Japan; d Faculty of Science and Technology, Oita University Oita 870-1192 Japan; e Research Institute for Electronic Science, Hokkaido University Sapporo 001-0020 Japan; f Department of Chemistry, Graduate School of Advanced Science and Engineering, Hiroshima University 1-3-1, Kagamiyama Higashi-hiroshima 739-8526 Japan; g Faculty of Advanced Science and Technology, Kumamoto University Kumamoto 860-8555 Japan

## Abstract

A novel flexible one-dimensional coordination polymer [Cu_2_(3-OH-bza)_3_(AcO)(pyr)] (1, 3-OH-bza = 3-hydroxybenzoate, AcO = acetate, pyr = pyrimidine) was found to adsorb water at rates that are influenced by the nature of coexisting gases. Upon exposure to a flow of water vapor containing gas, 1 displays a H_2_O adsorption rate that is decelerated to a greater extent by CO_2_ than by N_2_. Key to this phenomenon is the observation that 1 undergoes a structural change upon reversible and selective adsorption of H_2_O. This finding serves as the basis of a new strategy for designing porous materials for highly efficient separation and storage applications.

## Abstract 2

A flexible one-dimensional coordination polymer was found to show a H_2_O adsorption rate that is decelerated to a greater extent by CO_2_ than by N_2_.

## Graphical abstract

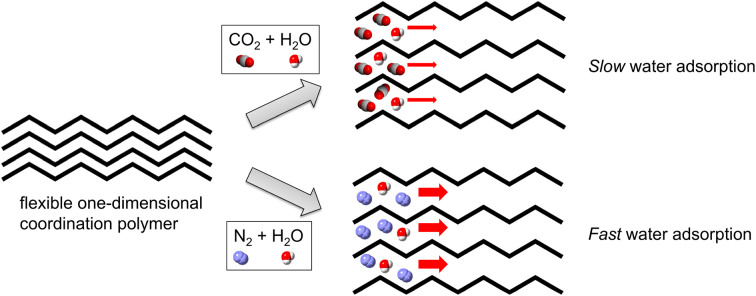

## Introduction

Porous materials exhibit interesting properties that enable their practical use in storage, mixture separation, catalysis, and ion exchange applications. Recently, metal–organic frameworks (MOFs) and porous coordination polymers (PCPs) have attracted attention as next-generation porous materials because of their high porosity, high structural designability, versatility and flexibility.^1–3^ A variety of properties of MOFs/PCPs have been thoroughly investigated, the results of which have demonstrated that their storage and mixture separation capabilities excel those of other porous materials.^4–10^

Several key parameters need to be considered when designing excellent storage and separation materials based on MOFs/PCPs, with adsorption rate being among the most important in the context of practical applications. Breakthrough experiments are often used to evaluate the adsorption and separation performances of materials under gas flow conditions. In these assessments, MOFs/PCPs need to be shaped because their use in fine particle form causes a large pressure drop. However, shaping MOFs/PCPs often significantly affects adsorption rates.^11,12^ On the other hand, rapid adsorption by these materials causes a decrease in cycle times, resulting in an increased throughput.^13^ In this regard, materials that operate using kinetic (or diffusion rate) separation are more efficient than those that rely on equilibrium separation.

Several studies have been conducted to develop approaches that increase and control the adsorption rates of MOFs/PCPs. For example, Vogel, Watanabe, *et al.* described a structurally hierarchal MOF that displays rapid gas adsorption. Specifically, these workers observed that a pellet packed with supraparticles of the zeolitic imidazolate framework-8 (ZIF-8) undergoes N_2_ adsorption at a rate that is 30 times faster than that of an unstructured ZIF-8 powder pellet.^11^ In another effort, Long *et al.* systematically investigated the CO_2_ adsorption kinetics of the three-dimensional (3D) MOF, [Mg_2_(dobpdc)] (dobpdc = 4,4′-dioxidobiphenyl-3,3′-dicarboxylate) and its diamine-appended derivatives. The findings show that the CO_2_ adsorption rates are enhanced and induction periods are decreased as the temperature decreases.^14^ Moreover, we have recently demonstrated that coating surfaces of MOF particles with a glassy nonporous coordination polymer (g-NCP) can be employed to alter adsorption rates. Specifically, we observed that the 3D MOF, [Cu_2_(pzdc)_2_(pyz)] (CPL-1, pzdc = 2,3-pyrazinedicarboxylate and pyz = pyrazine),^15,16^ has similar rates for adsorption of CO_2_, N_2_O and C_2_H_4_, whereas composites composed of this MOF and g-NCP, [Cu(bib)_2.5_]·2NTf_2_ (bib = 1,4-bisimidazole butane and NTf_2_ = bis(trifluoromethylsulfonyl)amide), display clearly different rates for adsorption of these gases. Differences in these materials are a consequence of the occurrence of gas diffusion within the g-NCP of the composite [Cu_2_(pzdc)_2_(pyz)]/g-NCP through a solution-diffusion mechanism.^17^

Most previous studies aimed at increasing and controlling adsorption rates, including those described above, have focused on changing the micro- or macrostructures of MOFs. In the current investigation described below, we developed an MOF in which the H_2_O adsorption rate is governed by the nature of coexisting gases. Specifically, the novel flexible one-dimensional (1D) coordination polymer [Cu_2_(3-OH-bza)_3_(AcO)(pyr)] (1, 3-OH-bza = 3-hydroxybenzoate, AcO = acetate, pyr = pyrimidine) adsorbs H_2_O when exposed to not only pure H_2_O vapor but also when exposed to flows of carrier gas (N_2_ and CO_2_) containing water vapor. Also, water adsorption promotes a structural change of 1. Measurements have shown that the rate of H_2_O adsorption under flow conditions is higher when N_2_ rather than CO_2_ is the carrier gas because of a difference in the diffusion coefficient of the binary gas system.

## Results and discussion

**Fig. 1:**
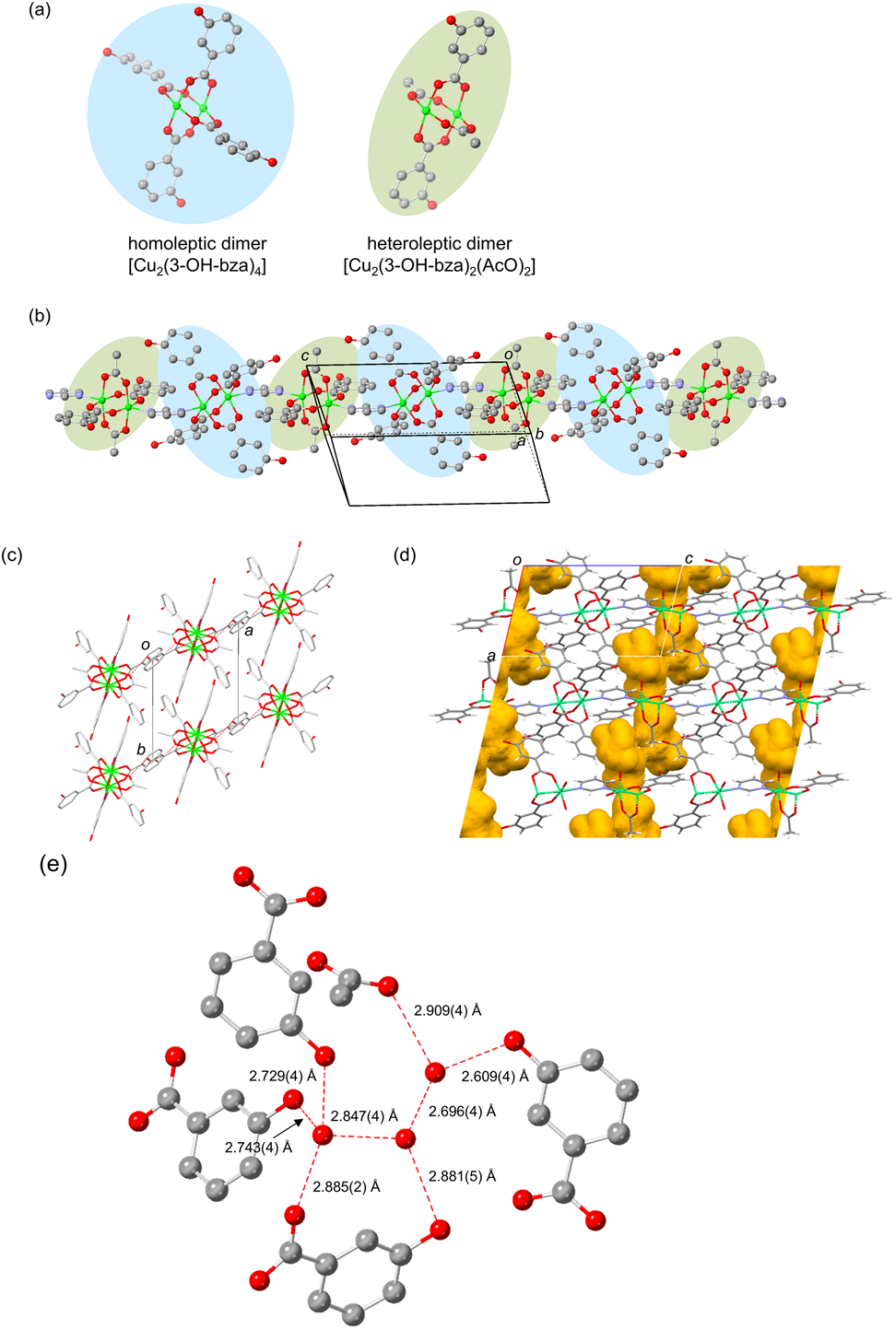
Crystal structure of 1·3H_2_O. (a) Two types of contributing paddlewheel dimers forming (b) a 1D zigzag chain structure. (c) Packing of the chains, (d) one-dimensional channel structure, and (e) hydrogen bonds involving guest water molecules. The green, blue, gray, and red represent copper, nitrogen, carbon, and oxygen, respectively. Hydrogen atoms are omitted for clarity except in (d). Red dashed lines represent hydrogen bonds.

[Cu_2_(3-OH-bza)_3_(AcO)(pyr)]·3H_2_O (1·3H_2_O) was prepared by the reaction of Cu(AcO)_2_·H_2_O, 3-hydroxybenzoic acid (3-OH-Hbza), and pyr in a solution of H_2_O and MeOH. X-ray crystallographic analysis (Fig. 1 and Table S1) shows that 1·3H_2_O has an unprecedented 1D chain structure composed of paddlewheel dimers. In contrast to most 1D coordination polymers that are created from one type of paddlewheel dimer,^18–20^1·3H_2_O contains an alternating array of two types of paddlewheel dimers, [Cu_2_(3-OH-bza)_4_] and [Cu_2_(3-OH-bza)_2_(AcO)_2_] (Fig. 1(a) and (b)). Furthermore, [Cu_2_(3-OH-bza)_2_(AcO)_2_] is a heteroleptic dimer containing mixed carboxylate groups. It is interesting to note that examples of 1D coordination polymers composed of heteroleptic dimers are limited, although a new method to synthesize heteroleptic paddlewheel dimers has been recently described by Miyasaka *et al.*^21^ The two types of dimers in 1·3H_2_O are alternately bridged by pyr to form a 1D zigzag chain oriented along the *c* axis (Fig. 1(b)). The 1D chains aggregate through COO⋯HO hydrogen bonding interactions (O⋯O distance = 2.750(4) Å, Fig. 1(c)) to form a porous crystal with one-dimensional channels aligned along the *a*-axis (Fig. 1(d) and S1). The amount of void space in the structure was calculated using Mercury software to be 11%. Guest H_2_O molecules are present in these channels positioned by rich hydrogen bonding interactions not only between the H_2_O molecules but also between the H_2_O and COO/OH groups of the carboxylate ligands (Fig. 1(e)).

**Fig. 2:**
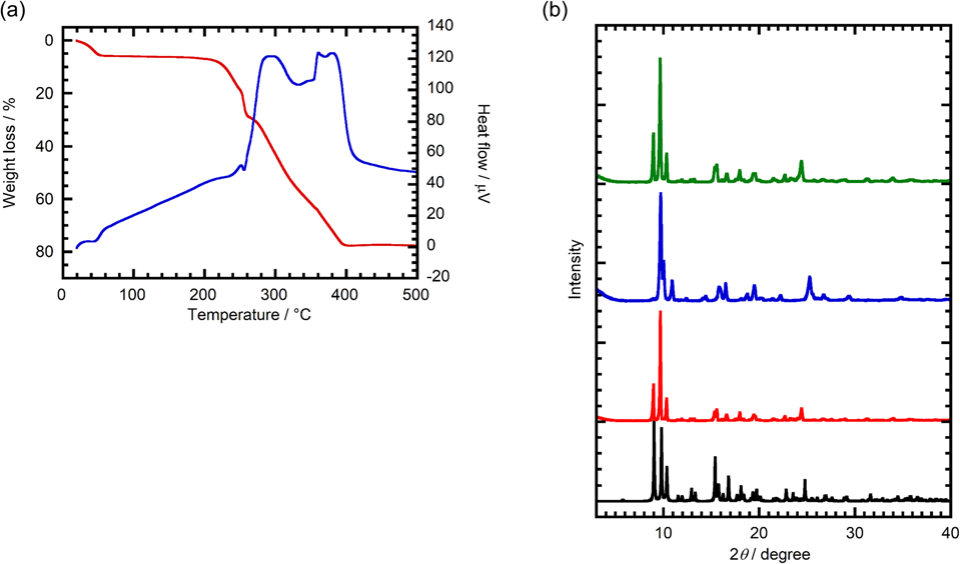
(a) TG (red)-DTA (blue) curves of 1·3H_2_O. (b) Simulated PXRD pattern of 1·3H_2_O (black), observed PXRD patterns of as-synthesized 1·3H_2_O (red), 1 obtained after heating of 1·3H_2_O at 100 °C under vacuum (blue), and 1·3H_2_O obtained after H_2_O exposure to dehydrated 1 (green).

The thermal properties of 1·3H_2_O were evaluated using a thermogravimetric-differential thermal analysis (TG-DTA) measurement. As shown in Fig. 2(a), guest H_2_O molecules in the material are released in the range of room temperature to *ca.* 50 °C. The weight loss at 50 °C is 5.4%, corresponding to 2.2 mol of H_2_O per 1 mol of 1. The weight loss, which is slightly less than 3 mol per 1 mol calculated from analysis of the crystal structure of 1·3H_2_O, is caused by gradual desorption of water in air. Desolvated 1 formed by H_2_O release is stable up to *ca.* 220 °C.

The attenuated total reflection (ATR)-infrared (IR) spectrum of desolvated 1 was found to contain only an O–H vibration band associated with 3-OH-bza ligands, supporting the conclusion that all guest H_2_O molecules are released during thermally promoted dehydration (Fig. S4(a)).

Powder X-ray diffraction (PXRD) analysis was employed to evaluate the structure of 1 after H_2_O removal. Desolvated 1 displays a PXRD pattern that differs from both the simulated and real patterns of 1·3H_2_O (Fig. 2(b)), suggesting that crystal-to-crystal structural transformation takes place during the release of H_2_O. Finally, exposure of desolvated 1 to H_2_O causes regeneration of the hydrated form 1·3H_2_O (Fig. 2(b)), revealing that H_2_O adsorption/desorption occurs reversibly in conjunction with a structural change.

**Fig. 3:**
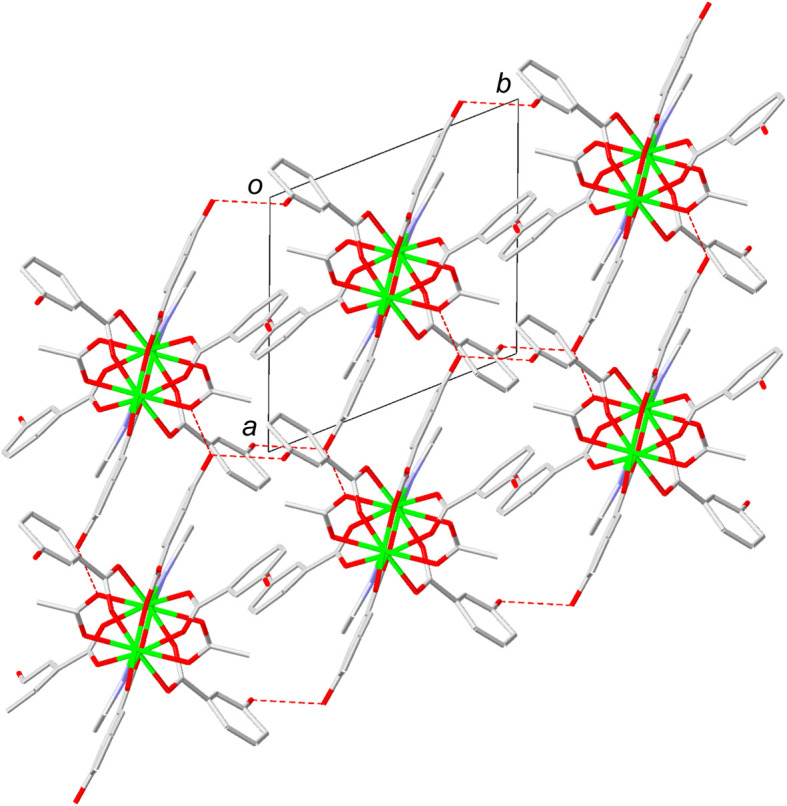
EDA-derived packing structure of chains in dehydrated 1. The green, blue, grey, and red colors represent copper, nitrogen, carbon, and oxygen, respectively. Hydrogen atoms are omitted for clarity. Red dashed lines represent hydrogen bonds.

We succeeded in carrying out structural characterization of desolvated 1 using electron diffraction analysis (EDA). Data obtained from EDA experiments show that after the release of H_2_O molecules, 1 retains its 1D zigzag chain structure (Fig. S2). However, the pattern of interchain hydrogen-bonding interactions is significantly changed upon dehydration. Specifically, in 1·3H_2_O, the interchain hydrogen bonds are oriented in the *ac* plane (Fig. 1(c)), while those in dehydrated 1 are aligned in a 3D manner presumably for crystal structural stabilization (Fig. 3). Thus, reorganization of the hydrogen bonding network in 1·3H_2_O takes place during dehydration to promote more dense packing of the chains creating a very small void space of 2% (Fig. S3). Also, the purity of the sample of 1 produced by thermal treatment of 1·3H_2_O was confirmed using PXRD. The cell parameters arising from Le Bail fitting for the PXRD pattern of the desolvated 1 agree well with those obtained employing EDA (Fig. S7), indicating that the sample includes only a single structure shown in Fig. 3.

**Fig. 4:**
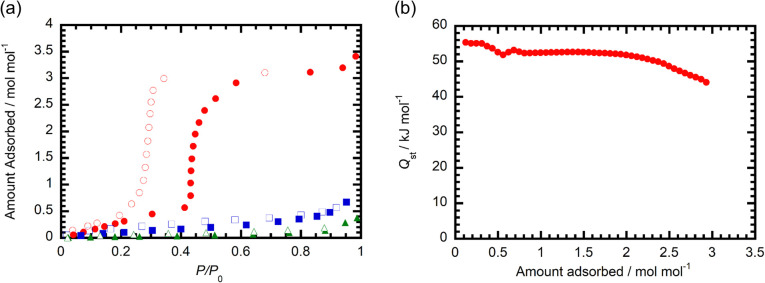
(a) H_2_O (red circles, 298 K), MeOH (blue squares, 288 K), and EtOH (green triangle, 298 K) adsorption (filled symbols)/desorption (open symbols) isotherms of 1. (b) Isosteric heat of water adsorption for 1.

Experiments were conducted to assess the detailed H_2_O adsorption properties of desolvated 1. In Fig. 4(a) are given the H_2_O, MeOH and EtOH adsorption/desorption isotherms of this material. The results indicate that a gradual increase in the amount of H_2_O adsorbed occurs in the *P*/*P*_0_ range from 0 to 0.41, and that the total amount of H_2_O adsorbed reaches 0.57 mol mol^−1^. Above *P*/*P*_0_ = 0.41, a sudden increase in H_2_O adsorption takes place reaching a saturated state of *ca.* 3 mol mol^−1^, which is consistent with formation of 1·3H_2_O. In the desorption process, 1·3H_2_O undergoes a rapid decrease in the amount of adsorbed H_2_O below *P*/*P*_0_ = 0.34, and the adsorption and desorption isotherms have a large hysteresis. These observations are consistent with the PXRD results indicating that 1 undergoes reversible H_2_O adsorption/desorption along with an accompanying structural change that can be described as H_2_O gated sorption. The results of experiments aimed at elucidating adsorption selectivity suggest that 1 adsorbs small amounts of not only N_2_ (77 K) and CO_2_ (195 and 298 K) gas (Fig. S8 and S9) but also MeOH and EtOH vapor (Fig. 3(a)). Overall, these observations indicate that 1 has high selectivity for H_2_O over other small molecules.

We next estimated the isosteric heat of adsorption (*Q*_st_) of 1 for H_2_O using isotherms obtained at various temperatures (Fig. S10). The *Q*_st_ values for 1 before and after rapid increases in adsorption amounts were found to be 52–55 and 44–53 kJ mol^−1^ (Fig. 3(b)), which are larger than the latent energy of water (40.7 kJ mol^−1^). The *Q*_st_ value of 53 kJ mol^−1^ determined after a rapid increase in the amount of adsorbed H_2_O is slightly higher than that found earlier for [Zr_6_O_4_(OH)_4_(MTB)_2_(HCOO)_4_(H_2_O)_2_] (MOF-841, MTB = 4,4′,4′′,4′′′-methanetetrayltetrabenzoate) (50 kJ mol^−1^).^22^

**Fig. 5:**
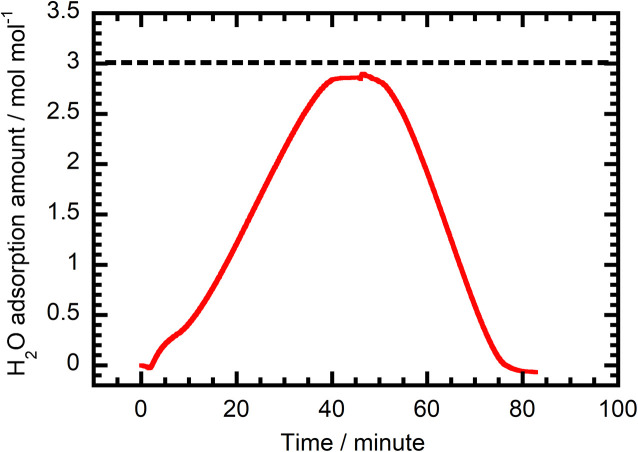
Time dependence of the amount of H_2_O adsorbed by 1 under humidified N_2_ (0–46 min) and dry N_2_ (46–83 min) at 300 K.

The H_2_O adsorption/desorption behaviour of 1 was also evaluated using TG-DTA under humidified and dry N_2_ conditions. As the plot in Fig. 5 shows, H_2_O adsorption at 300 K reaches a plateau after 40 min, at which the amount of H_2_O adsorbed is 2.86 mol mol^−1^. This value is very close to the *ca.* 3 mol mol^−1^ value arising from the H_2_O adsorption isotherm. These results suggest that 1 adsorbs H_2_O almost exclusively even under mixed N_2_–H_2_O flow conditions. After switching from wet to dry N_2_ gas, the amount of adsorbed H_2_O decreases to zero at 80 min. When the carrier gas is CO_2_, similar H_2_O content changes take place (humidified CO_2_ flow reaching 2.86 mol mol^−1^ and dry flow back to zero, Fig. S12), suggesting that the nature of the carrier gas has no effect on the amount of H_2_O adsorbed.

**Fig. 6:**
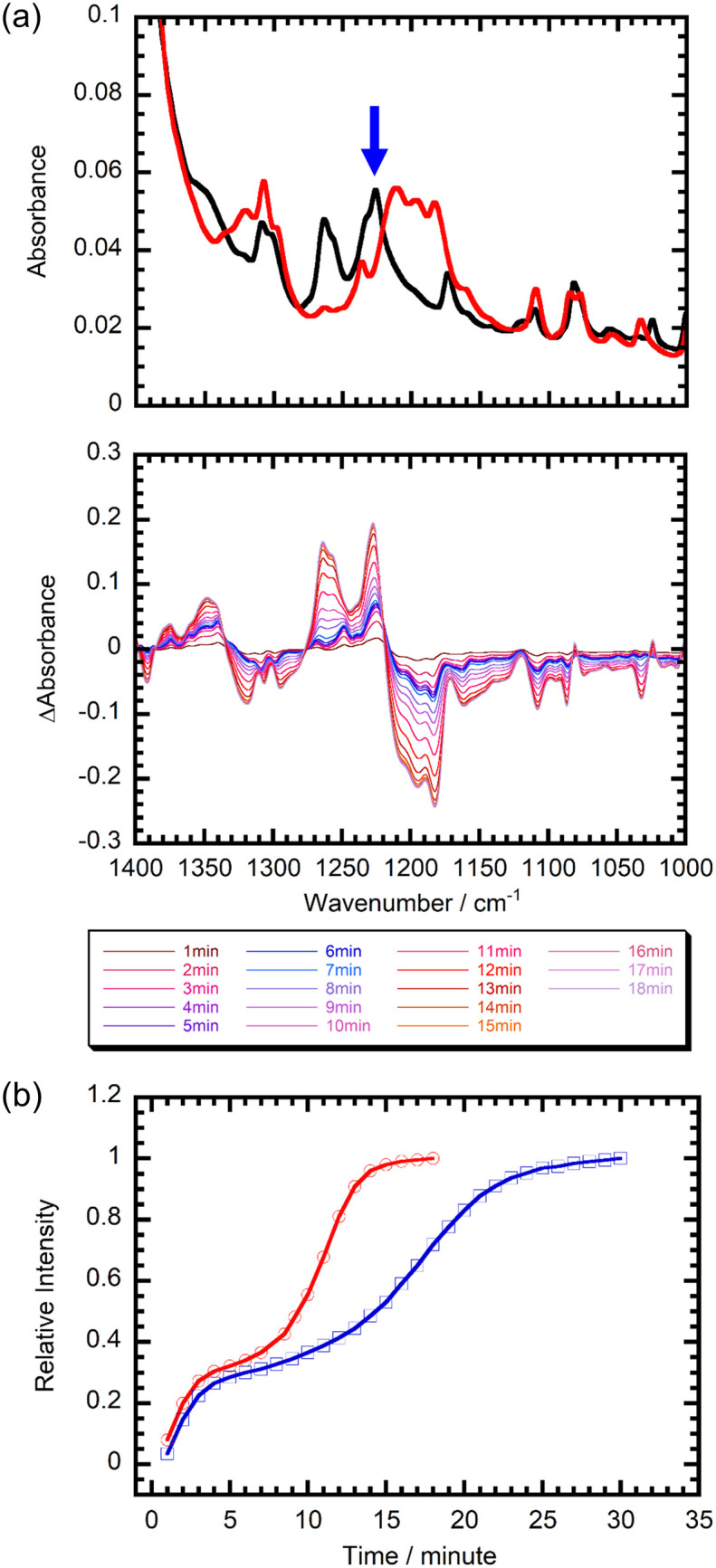
(a) ATR-IR spectra of 1 (red) and 1·3H_2_O (black) and difference IR spectra of 1 under wet N_2_ at 303 K over 1 min intervals with the humid N_2_ gas flow beginning at 0 min. (b) Time dependence of relative intensity for a peak at 1227 cm^−1^ (blue arrow in (a) indicates the wavenumber monitored) under humid N_2_ (red) and humid CO_2_ (blue) at 303 K in 1.

We also determined the rates of H_2_O adsorption by 1 using IR spectroscopy rather than TG-DTA because of the difficulties in controlling temperatures near room temperature and performing rate measurements inside the TG-DTA chamber. Comparison of ATR-IR spectra of 1·3H_2_O and 1 indicates that dramatic differences exist in the 1150–1300 cm^−1^ range (Fig. S4(b)), which are associated with C–O (hydroxyl oxygen) vibration bands of 3-OH-bza ligands (Fig. S5). These observations are consistent with the results of the crystal structure analysis that suggest that dehydration alters the hydrogen bonding patterns of the OH groups. Therefore, this wavelength range was employed to monitor the rates of H_2_O adsorption by 1. Inspection of the spectra in Fig. 6(a) shows that dramatic changes in peak intensities occur when 1 is subjected to a humid N_2_ gas flow because of structural changes promoted by H_2_O adsorption. The time course for changes in the intensity of the peak at 1227 cm^−1^ given in Fig. 6(b) indicates that the adsorption process reaches saturation after 15 min. Surprisingly, the rate of H_2_O adsorption by 1 is carrier gas dependent. Specifically, although the spectral changes promoted by subjecting 1 to a flow of humidified CO_2_ are the same as those brought about by humid N_2_ (Fig. S13), they occur more slowly than in the former experiment (especially, after 7 min) to reach the saturation point after 25 min (Fig. 6(b)). The adsorption rates (*k*_N_2_-ad_ and *k*_CO_2_-ad_) calculated using the time dependency data were found to be 0.11 and 0.055 min^−1^ for wet N_2_ and CO_2_ gases, respectively (Fig. S14). Notably, H_2_O desorption under dry N_2_ and CO_2_ flows occurs at similar rates (*k*_N_2_-de_ = 0.80 and *k*_CO_2_-de_ = 0.85 min^−1^) and more rapidly than adsorption of H_2_O (Fig. S15–S18).

To confirm the generality of this phenomenon, similar water adsorption experiments were performed on another flexible coordination polymer. The two-dimensional (2D) [Cu(CF_3_SO_3_)_2_(bpp)_2_] (bpp = 1,3-bis(4-pyridyl)propane) shows reversible H_2_O adsorption/desorption with temporary expansion of the 2D layer.^23^ This coordination polymer adsorbs *ca.* 1 mol mol^−1^ of H_2_O at 298 K (ref. 23) but adsorbs only a small amount of CO_2_ at the same temperature (Fig. S11). The ATR-IR spectrum of [Cu(CF_3_SO_3_)_2_(bpp)_2_] contains vibration bands for CF_3_SO_3_ anions in the 1000–1300 cm^−1^ range (Fig. S19).^23^ Analysis of the time-dependence of the relative intensity of the IR peak at 1163 cm^−1^ (Fig. S19–S21) indicates that the rate of H_2_O adsorption under a humid CO_2_ gas flow is slower than that under a humid N_2_ gas flow (*k*_N2-ad_ and *k*_CO2-ad_ = 0.39 and 0.29 min^−1^ for wet N_2_ and CO_2_ gases, respectively, Fig. S22). However, the difference in the adsorption rate, calculated using the time dependency data (*k*_N_2_-ad_/*k*_CO_2_-ad_ = 1.34), is smaller than that observed for 1 (*k*_N_2_-ad_/*k*_CO_2_-ad_ = 2.00), indicating that the degree of a coexisting guest effect depends on nature of the structure. On the other hand, the desorption rates are almost the same (*k*_N_2_-de_ and *k*_CO_2_-de_ = 0.42 and 0.41 min^−1^) regardless of the type of coexisting gas (Fig. S23–S26).

Several possible sources for the carrier gas dependence of the rate of H_2_O adsorption were considered. First, we realized that CO_2_ has high solubility in H_2_O, and that dissolution results in the formation of carbonic acid (H_2_CO_3_) and its conjugate bases (HCO_3_^−^ and CO_3_^2−^). Moreover, Kitagawa, Onoe, *et al.* reported that CO_3_^2−^ anions form in the specific sub-nm space of a 1D uneven-structured C_60_ polymer film subjected to CO_2_ and H_2_O at room temperature.^24^ While H_2_CO_3_ and CO_3_^2−^ display bands at *ca.* 1180 and 1363 cm^−1^, respectively,^24^ these researchers observed a band at only 1370 cm^−1^ in the IR spectrum of the C_60_ polymer film after exposure to atmospheric air (*ca.* 14% humidity), suggesting the presence of CO_3_^2−^ anions.

**Fig. 7:**
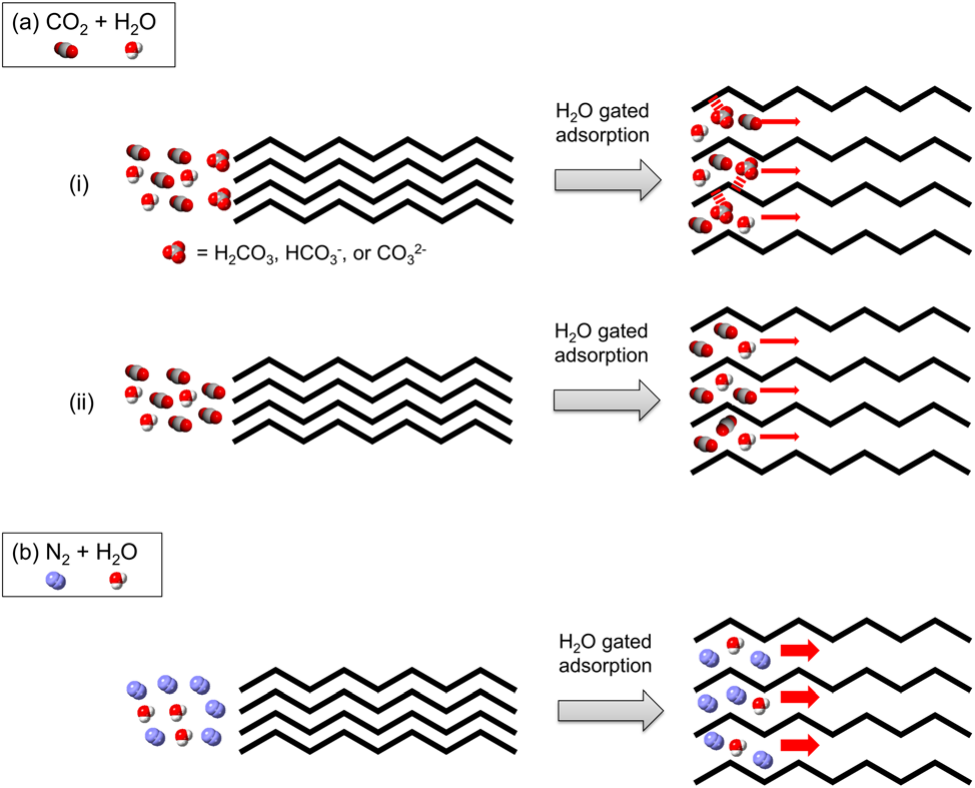
Possible source of the coexisting gas dependency of the H_2_O adsorption rate. (a) Temporary formation of H_2_CO_3_/HCO_3_^−^/CO_3_^2−^ (i) and temporary occupancy of CO_2_ and H_2_O in the pores (ii) under humid CO_2_ flow conditions. (b) Temporary occupancy of N_2_ and H_2_O in the pores under wet N_2_ flow conditions.

It is possible that H_2_CO_3_, HCO_3_^−^ and CO_3_^2−^, formed transiently during H_2_O adsorption under a humid CO_2_ gas flow, decrease the H_2_O adsorption rate owing to a blocking effect caused by strong H_2_CO_3_/HCO_3_^−^/CO_3_^2−^-interactions with framework sites in 1 (Fig. 7(a(i)). To determine if the formation of H_2_CO_3_, HCO_3_^−^ and CO_3_^2−^ occurs under a humid CO_2_ flow, the 1000–1400 cm^−1^ region in IR spectra of 1 under humid CO_2_ and humid N_2_ flows was analyzed. However, no differences were found to exist in spectra obtained using both flows (Fig. 6(a) and S13).

Next, we focused on diffusion coefficients for binary gas systems because it is known that this parameter varies with the type of coexisting gas in a mixture. For example, the diffusion coefficient of the CO_2_–H_2_O binary (equimolar mixture) system at 293.15 K is 0.162 cm^2^ s^−1^, a value that is considerably lower than that of the N_2_–H_2_O binary system (0.242 cm^2^ s^−1^ at 293.15 K).^25^ Therefore, the greater propensity of CO_2_ to decelerate water diffusion is the likely cause of the lower rate of H_2_O adsorption by 1 under a CO_2_–H_2_O flow compared to a N_2_–H_2_O flow (Fig. 7(a(ii)) and (b)).

**Fig. 8:**
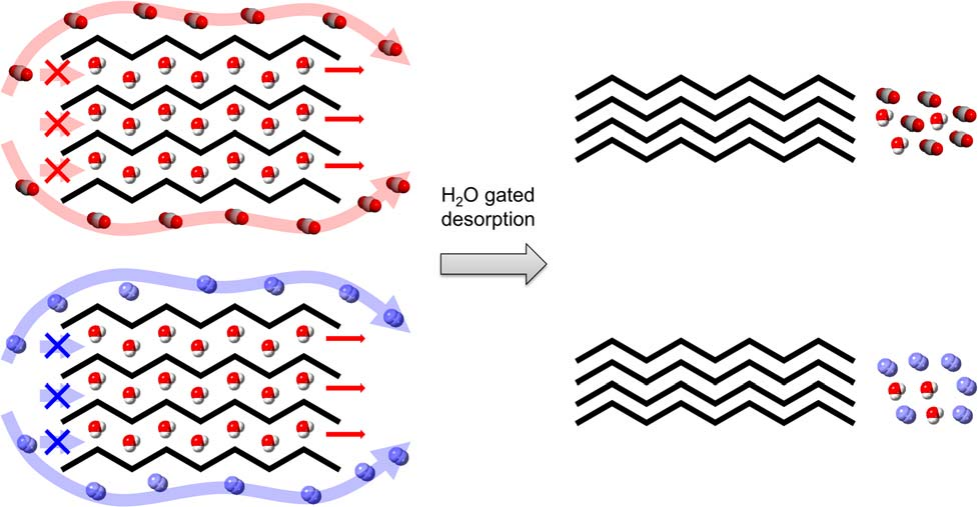
Scheme of the possible source of the coexisting gas independency of the H_2_O desorption rate.

On the other hand, a carrier gas independence of the H_2_O desorption rates was observed, suggesting that the H_2_O adsorption and desorption processes in the presence of coexisting guests are considerably different. During H_2_O desorption, CO_2_ and N_2_ do not diffuse through pores as shown in Fig. 8, which is the likely cause of the observed similar desorption rates under N_2_ and CO_2_ flows.

If correctly interpreted, this finding would be the first example of a coexisting guest effect on rates of adsorption of guests by materials. This proposal raises the question whether CO_2_ and/or N_2_ gases diffuse through pores of 1 (Fig. 7) along with H_2_O while not being adsorbed. Previously, we investigated the influence of co-adsorbates on CO_2_ gated sorption in flexible MOFs.^26^ We observed that [Zn_2_(DiP-bdc)_2_(dabco)] (DiP-bdc = 2,5-diisopropoxy-1,4-benzenedicarboxylate; dabco = 1,4-diazabicyclo[2.2.2]octane) displays gated sorption of CO_2_, C_2_H_6_ and C_3_H_8_ in conjunction with a structural change from a narrow pore (np) to a large pore (lp) form. In contrast, N_2_ and CH_4_ do not promote gate opening, which results in low adsorption of these gases. However, results of co-adsorption measurements demonstrated that after CO_2_ induced gate opening, CH_4_ co-adsorbs in the lp phase. In other words, CH_4_ enters pores of this MOF along with CO_2_. Based on these previous results, it is reasonable to propose that unlike H_2_O, CO_2_ and N_2_ occupy the pores only transiently. However, further investigations are needed to confirm that transient occupation in the pores occurs.

## Conclusions

In conclusion, we investigated features of H_2_O adsorption by the 1D coordination polymer [Cu_2_(3-OH-bza)_3_(AcO)(pyr)] (1) under gas flow conditions. The results of single-crystal X-ray analysis, EDA, and gas and vapor adsorption/desorption measurements show that the coordination polymer displays reversible and selective H_2_O adsorption in conjunction with a structural change. Kinetic analysis carried out by using *in situ* IR spectroscopy indicates that the rate of H_2_O adsorption under a humid CO_2_ gas flow is slower than that under a humid N_2_ gas flow. This trend is consistent with the diffusion coefficients of the respective binary gas systems. Specifically, the considerably lower diffusion coefficient of the CO_2_–H_2_O mixture compared to that of the corresponding N_2_–H_2_O mixture is responsible for the greater propensity of CO_2_ to decelerate water adsorption by 1. In addition, this coexisting guest effect was observed in another flexible 2D coordination polymer. While it is obvious that the diffusion coefficient of the binary gas system depends on the types of gases, it is noteworthy that the phenomenon was observed for gas flows within a very narrow (<1 nm) space. Therefore, this finding suggests that a new strategy exists to control gas/vapor adsorption rates of porous materials, and that the approach might be applicable to the development of efficient gas storage and separation materials.

## Author contributions

S. N. conceptualized the project. A. W., X. Z., Y. S., A. T., Y. H., K. T. and T. N. contributed to data collection and formal analyses. S. N., Y. K. and Y. H. set up and performed *in situ* IR spectra measurements. H. S. performed electron diffraction measurements and analyses. A. K. performed Le Bail fitting of the PXRD pattern. S. N. wrote the manuscript, and all the authors approved the final version.

## Conflicts of interest

There are no conflicts to declare.

## Supplementary Material

SC-016-D5SC01699A-s001

SC-016-D5SC01699A-s002

## Data Availability

CCDC 2428329 (1·3H_2_O) and 2428330 (1) contain the supplementary crystallographic data for this paper.^27*a*,*b*^ Supplementary information is available. See DOI: https://doi.org/10.1039/d5sc01699a.

## References

[cit1] Kitagawa S., Kitaura R., Noro S. (2004). Angew. Chem., Int. Ed..

[cit2] Furukawa H., Cordova K. E., O'Keeffe M., Yaghi O. M. (2013). Science.

[cit3] Carlucci L., Ciani G., Proserpio D. M., Mitina T. G., Blatov V. A. (2014). Chem. Rev..

[cit4] Peng Y., Krungleviciute V., Eryazici I., Hupp J. T., Farha O. K., Yildrim T. (2013). J. Am. Chem. Soc..

[cit5] Suresh K., Aulakh D., Purewal J., Siegel D. J., Veenstra M., Matzger A. J. (2021). J. Am. Chem. Soc..

[cit6] Lu W., Jayasinghe D. D. A., Schröder M., Yang S. (2024). Acc. Mater. Res..

[cit7] Nguyen T. T. T., Lin J.-B., Shimizu G. K. H., Rajendran A. (2022). Chem. Eng. J..

[cit8] Su Y., Otake K., Zhang J.-J., Horike S., Kitagawa S., Gu C. (2022). Nature.

[cit9] Vervoorts P., Schneemann A., Hante I., Pirillo J., Hijikata Y., Toyao T., Kon K., Shimizu K., Nakamura T., Noro S., Fischer R. A. (2020). ACS Appl. Mater. Interfaces.

[cit10] Tulchinsky Y., Hendon C. H., Lomachenko K. A., Borfecchia E., Melot B. C., Hudson M. R., Tarver J. D., Korzyński M. D., Stubbs A. W., Kagan J. J., Lamberti C., Brown C. M., Dincă M. (2017). J. Am. Chem. Soc..

[cit11] Fujiwara A., Wang J., Hiraide S., Götz A., Miyahara M. T., Hartmann M., Zubiri B. A., Spiecker E., Vogel N., Watanabe S. (2023). Adv. Mater..

[cit12] Chen Y., Huang X., Zhang S., Li S., Cao S., Pei X., Zhou J., Feng X., Wang B. (2016). J. Am. Chem. Soc..

[cit13] Yang R. T. , Gas Separation by Adsorption Processes, Butterworth-Heinemann, Boston, 1987, ch. 5, pp. 141–200

[cit14] Martell J. D., Milner P. J., Siegelman R. L., Long J. R. (2020). Chem. Sci..

[cit15] Kondo M., Okubo T., Asami A., Noro S., Yoshitomi T., Kitagawa S., Ishii T., Matsuzaka H., Seki K. (1999). Angew. Chem., Int. Ed..

[cit16] Kitaura R., Matsuda R., Kubota Y., Kitagawa S., Takata M., Kobayashi T. C., Suzuki M. (2005). J. Phys. Chem. B.

[cit17] Zheng X., Kato M., Uemura Y., Matsumura D., Yagi I., Takahashi K., Noro S., Nakamura T. (2023). Inorg. Chem..

[cit18] Takamizawa S., Nakata E., Akatsuka T., Miyake R., Kakizaki Y., Takeuchi H., Maruta G., Takeda S. (2010). J. Am. Chem. Soc..

[cit19] Kosaka W., Zhang J., Watanabe Y., Miyasaka H. (2022). Inorg. Chem..

[cit20] Noro S., Meng Y., Suzuki K., Sugiura M., Hijikata Y., Pirillo J., Zheng X., Takahashi K., Nakamura T. (2021). Inorg. Chem..

[cit21] Sekine Y., Kosaka W., Kano H., Dou C., Yokoyama T., Miyasaka H. (2016). Dalton Trans..

[cit22] Furukawa H., Gándara F., Zhang Y.-B., Jiang J., Queen W. L., Hudson M. R., Yaghi O. M. (2014). J. Am. Chem. Soc..

[cit23] Fukuhara K., Noro S., Sugimoto K., Akutagawa T., Kubo K., Nakamura T. (2013). Inorg. Chem..

[cit24] Nakaya M., Kitagawa Y., Watanabe S., Teramoto R., Era I., Nakano M., Onoe J. (2021). Adv. Sustainable Syst..

[cit25] CRC Handbook of Chemistry and Physics, ed. David R. Lide, CRC Press, 88th edn, 2008

[cit26] Schneemann A., Takahashi Y., Rudolf R., Noro S., Fischer R. A. (2016). J. Mater. Chem. A.

[cit27] (a) Wang A. Zheng X. , Saito Y., Tateishi A., Huang Y., Kamiya Y., Sato H., Kondo A., Takahashi K., Nakamura T. and Noro S., CCDC 2428329: Experimental Crystal Structure Determination, 2025, 10.5517/ccdc.csd.cc2mhw67

