# Supplementary material for: Coexisting gases regulate the rates of water adsorption by a flexible one-dimensional coordination polymer
Source: Chem Sci. 2025 Aug 27;16(39):18135–40. doi: 10.1039/d5sc01699a (PMC12418625; doi:10.1039/d5sc01699a)
Supplement: SC-016-D5SC01699A-s001 [file SC-016-D5SC01699A-s001.pdf]

Supplementary Information (SI) for Chemical Science.
This journal is © The Royal Society of Chemistry 2025

Coexisting gases regulate the rates of water adsorption by a flexible one-dimensional coordination polymer

AnQi Wang,[a] Xin Zheng,[ab] Yuki Saito,[ab] Arata Tateishi,[a] Yuan Huang,[b] Yuichi Kamiya,[b] Hiroyasu Sato,[c] Atsushi Kondo,[d] Kiyonori Takahashi,[ae] Takayoshi Nakamura[aef] and Shin-ichiro Noro*[ab]

[a]Graduate School of Environmental Science, Hokkaido University, Sapporo 060-0810, Japan

[b]Faculty of Environmental Earth Science, Hokkaido University, Sapporo 060-0810, Japan

[c]Rigaku Corporation, Akishima 196-8666, Japan

[d]Faculty of Science and Technology, Oita University, Oita 870-1192, Japan

[e]Research Institute for Electronic Science, Hokkaido University, Sapporo 001-0020, Japan

[f]Department of Chemistry, Graduate School of Advanced Science and Engineering, Hiroshima University, 1-3-1, Kagamiyama, Higashi-hiroshima 739-8526, Japan

Table of contents

**S1**	Experimental detail	**2-3**
**S2**	Crystallographic data	**4**
**S3**	Crystal structures	**5-6**
**S4**	IR spectroscopy	**7-8**
**S5**	Powder X-ray diffraction patterns	**9-10**
**S6**	Adsorption properties	**11-12**
**S7**	TG analysis under a moist gas flow	**13**
**S8**	IR spectroscopy under a moist gas flow	**14-24**
**S9**	References	**25**

S1. Experimental detail

Materials. All chemicals were purchased from commercial sources and used without purification. The two-dimensional flexible coordination polymer [Cu(CF_3SO_3)$_2$(bpp)$_2$] (bpp = 1,3-bis(4-pyridyl)propane) was synthesized according to the literature.[S1]

Synthesis of [Cu_2(3-OH-bza)$_3$(AcO)(pyr)]·3H_2O (1·3H_2O). 3-hydroxybenzoic acid (3-OH-Hbza, 276 mg, 2.00 mmol) was added to a mixed solution of H_2O and MeOH (10 mL + 10 mL) containing Cu(AcO)$_2$·H_2O (AcO = acetate, 200 mg, 1.00 mmol). Then, pyrimidine (pyr, 40 mg, 0.50 mmol) was added to the mixed solution. The obtained transparent blue solution was allows to stand at room temperature for 10-15 days, yielding green crystals. The crystals were filtered and dried at room temperature. Yield: 149 mg (44 %). Elemental analysis (%) calcd for $C_{27}H_{22}Cu_2N_2O_{11}$: C 47.86, H 3.27, N 4.13. Found: C 46.75, H 3.05, N 4.07.

Physical measurements. Elemental analysis (C, H, and N) was performed at the Global Facility Center, Hokkaido University, using an Elemental Analyzer CE440 (Exeter Analytical, Inc.). Attenuated total reflection (ATR)-Infrared (IR) spectra were recorded using an iS10 FT-IR spectrometer equipped with a GladiATRTM accessory (Thermo Fisher Scientific) with a resolution of 4 cm^{-1}. IR spectra under wet gas flow were recorded on a JASCO FT/IR-6100 equipped with an MCT detector with a resolution of 2 cm^{-1}. The sample was set on a Si plate, and the Si plate was placed in a quartz IR cell equipped with NaCl windows connected to a gas flow system. The sample was heated at 373 K under dry N_2 flow (50 mL·min^{-1}) for 15 min and then cooled to 303 K. After heating, tha gas was switched to a wet N_2 or CO_2 gas (50 mL·min^{-1}) with the humidity of 55 % at 303 K. Gas flow was controlled using a mass flow controller, calibrated with a soap membrane flowmeter each time we changed the gas used. Powder X-ray diffraction data were collected on a Bruker D2 Phase X-ray diffractometer with Ni-filtered Cu Kα radiation (λ = 1.5406 Å) at a voltage of 30 kV and a current of 10 mA. The Le Bail fitting analysis was performed using the EXPO2014 software.[S2] Thermogravimetric analyses were performed using a Rigaku Thermo Plus TG8120 in the temperature range from 298 to 773 K under an N_2 flow of 100 mL·min^{-1} at a heating rate of 10 K·min^{-1}. The weight changes under dry and wet gases were monitored using the Rigaku Thermo Plus TG8120. First, samples were activated under a dry N_2 or CO_2 gas flow of 100 mL·min^{-1} for at least 20 min. Then, a wet N_2 or CO_2 gas with a humidity of ca. 70 % was dosed into the apparatus. After no weight change was observed, a dry gas was flowed into the apparatus. Vapor and gas adsorption/desorption isotherms were measured using Microtracbel BELSORP-aqua and BELSORP-mini II

volumetric adsorption equipment, respectively. Before conducting isotherm measurements, the sample was heated at 373 K under reduced pressure (<10 Pa) for more than 24 h.

Single-crystal X-ray diffraction analysis. The single-crystal structural analysis of **1**$\cdot 3H_2O$ was performed at 173 K using a Rigaku Micro Max-007 HF diffractometer and a Pilatus 200 K detector with Cu Kα radiation (λ = 1.54184 Å). The initial structure was solved using SHELXT,[S3] and structural refinement was performed by full-matrix least-squares techniques on F^2 using SHELXL.[S4] Anisotropic refinement was applied to all atoms except hydrogen atoms. Olex2 was used as a graphical user interface.[S5]

Electron diffraction analysis. Continuous rotation 3D electron diffraction data were acquired using the dedicated electron diffractometer Rigaku XtaLAB Synergy-ED. Data acquisition was performed at ambient temperature with an electron wavelength of 0.0251 Å (200 kV). The data were processed using CrysAlisPro for ED, the structure was solved using SHELXT[S3] and subsequently refined using SHELXL[S4] in the crystallographic program suite Olex2.[S5]

S2. Crystallographic data

Table S1. Crystallographic data of **1**·3H_2O and **1**.

	**1**·3H_2O	**1**
Chemical Formula	[Cu_2(3-OH-bza)$_3$(AcO)(pyr)]·3H_2O	[Cu_2(3-OH-bza)$_3$(AcO)(pyr)]
Formula	$C_{27}H_{28}Cu_2N_2O_{14}$	$C_{27}H_{22}Cu_2N_2O_{11}$
Formula weight	731.59	677.57
Crystal system	Triclinic	Triclinic
Space group	*P*1-	*P*1-
Temperature / K	173	293
a / Å	9.9417(2)	9.462(3)
b / Å	10.7906(2)	9.915(3)
c / Å	15.9286(3)	15.687(3)
α / Å^3	98.869(2)	94.47(2)
β / Å^3	98.980(2)	94.64(2)
γ / Å^3	111.275(2)	111.54(3)
V / Å^3	1530.99(6)	1355.2(7)
Z	2	2
GOF on F^2	1.102	1.108
R_1 [$I > 2\sigma(I)$][a]	0.0435	0.2093
R_w [$I > 2\sigma(I)$][b]	0.1305	0.4185
CCDC number	2428329	2428330

[a] $R_1 = \sum||F_o| - |F_c||/\sum|F_o|$. [b] $R_w = [(\sum w(|F_o|^2 - |F_c|^2)^2)/\sum w(F_o^2)^2]^{1/2}$.

S3. Crystal structures

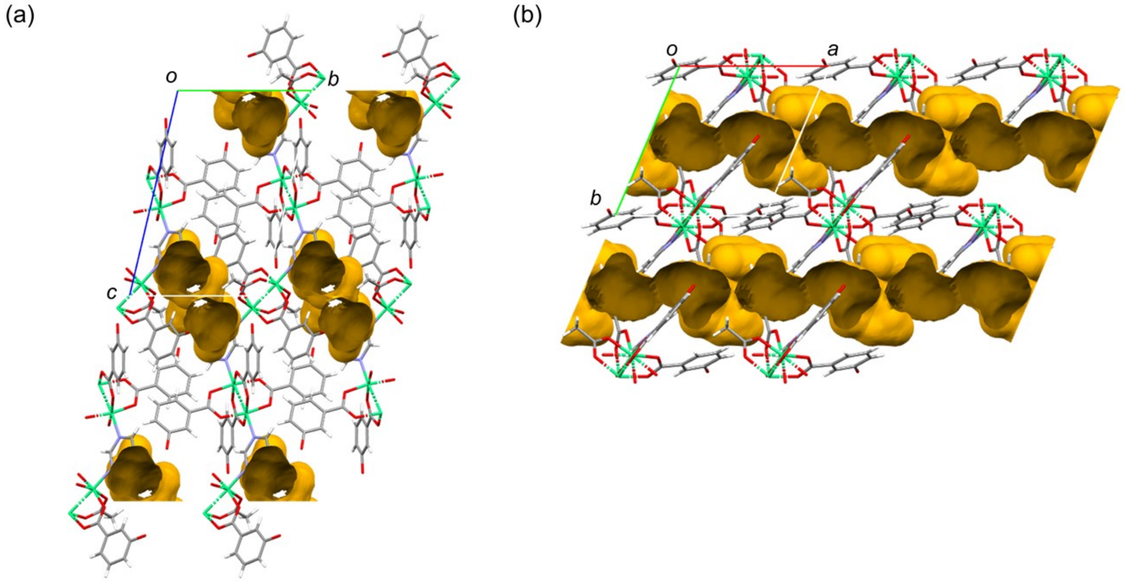

Fig. S1. 1D channel structure viewed along the (a) *a*- and (b) *c*-axes in **1**·3H_2O. The green, blue, gray, and red colors represent copper, nitrogen, carbon, and oxygen, respectively. The hydrogen atoms are omitted for clarity.

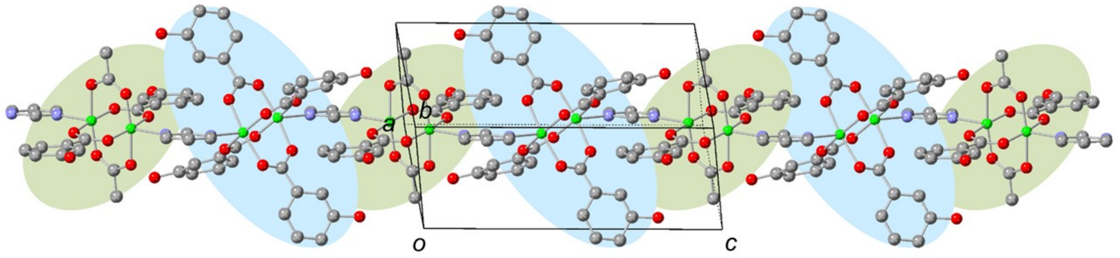

Fig. S2. 1D zigzag chain structure with the two types of paddlewheel dimers, [Cu_2(3-OH-bza)$_4$] (blue) and [Cu_2(3-OH-bza)$_2$(AcO)$_2$] (green), in **1**. The green, blue, gray, and red colors represent copper, nitrogen, carbon, and oxygen, respectively. The hydrogen atoms are omitted for clarity.

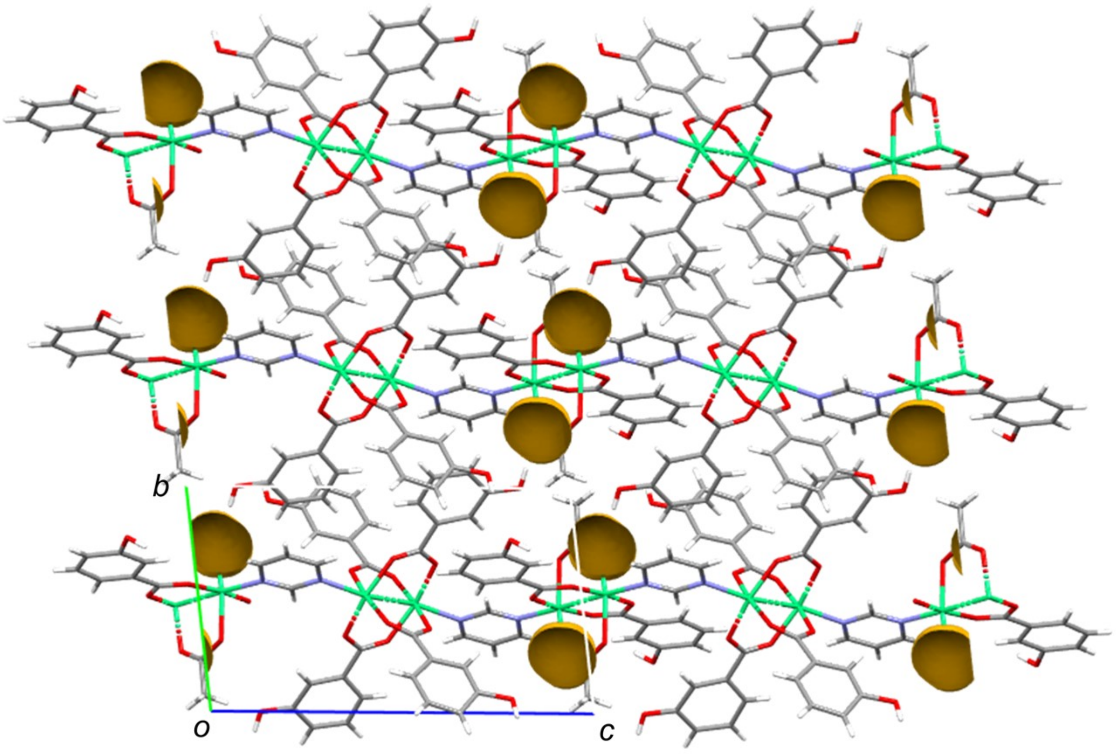

Fig. S3. Packing view of chains in **1** viewed along the *a*-axis. The void space is colored orange.

S4. IR spectroscopy

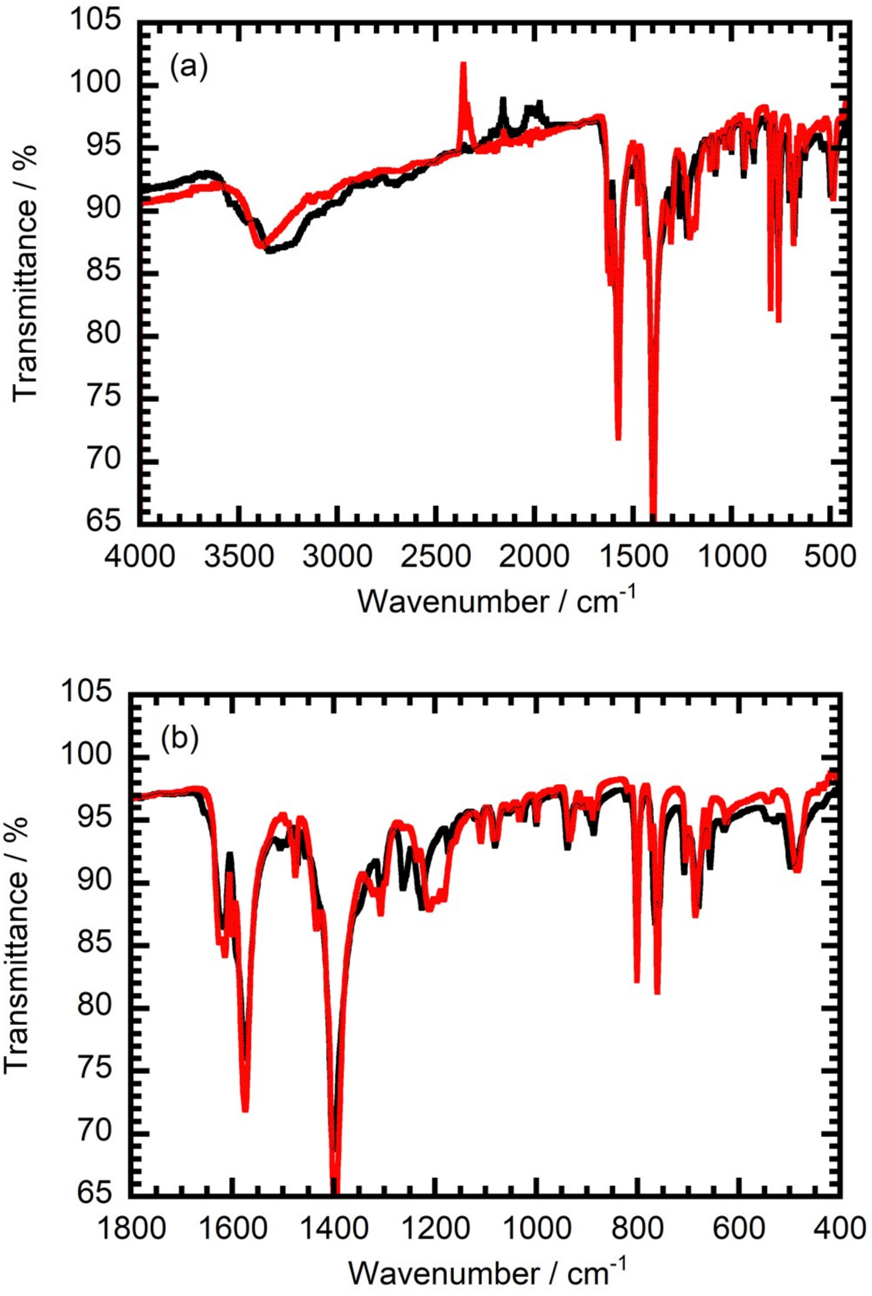

Fig. S4. ATR-IR spectra of **1**·3H_2O (black) and **1** (red) in the range of (a) 400-4000 and (b) 400-1800 cm^{-1}.

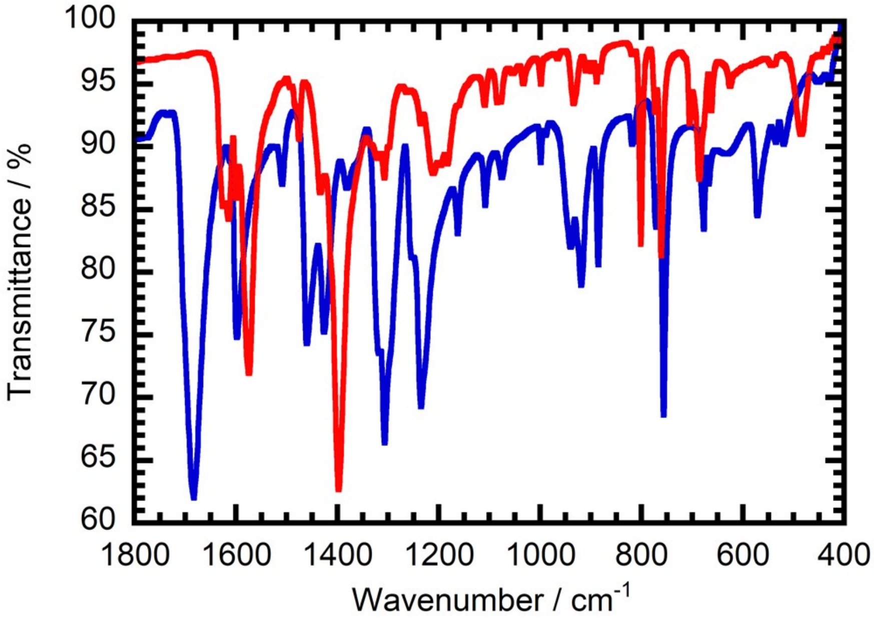

Fig. S5. ATR-IR spectra of **1** (red) and 3-OH-Hbza (blue).

S5. Powder X-ray diffraction patterns

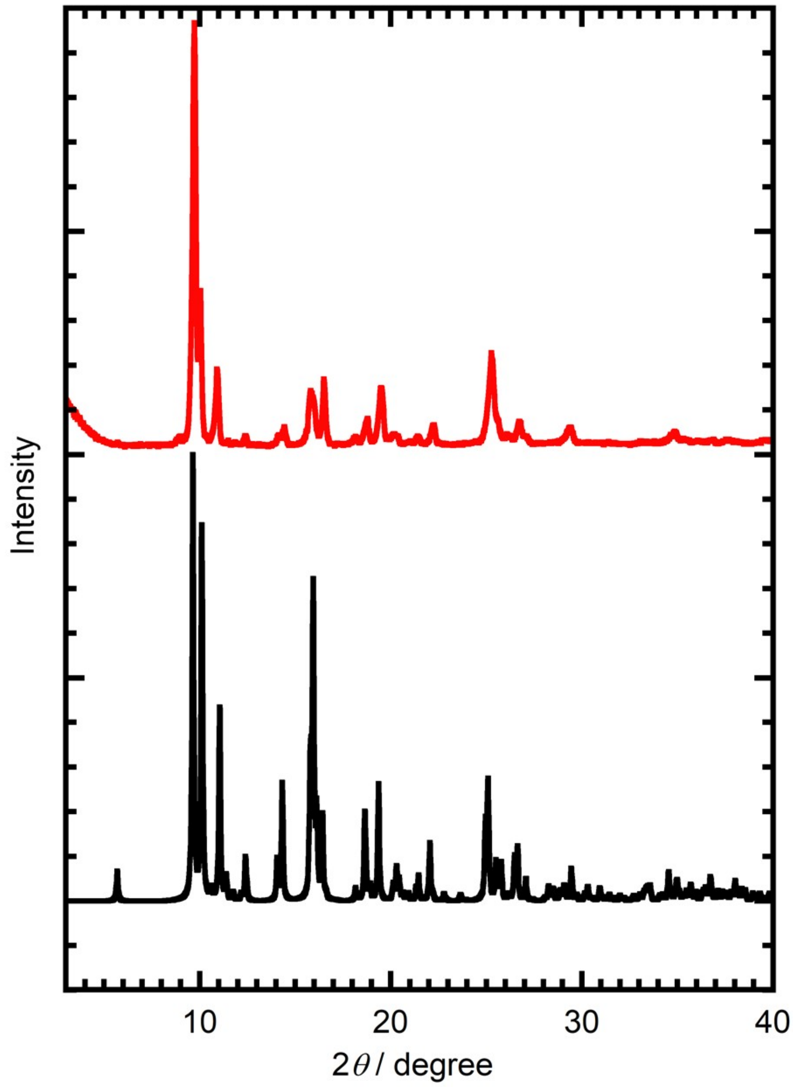

Fig. S6. Powder X-ray diffraction patterns of simulated **1** (black) and **1** (red).

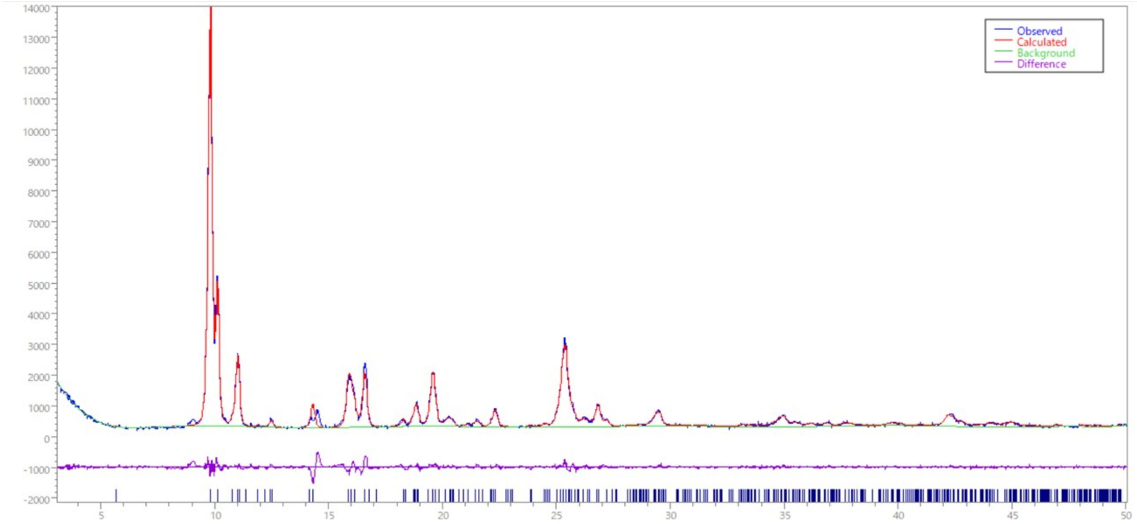

Fig. S7. The results of LeBail fitting analysis for the powder X-ray diffraction pattern of **1** at 298 K. The lattice parameters were refined to be a = 9.479(6) Å, b = 9.780(6) Å, c = 15.716(10) Å, α = 94.327(19) º, β = 94.725(15) º, γ = 111.778(10) º, V = 1339.4(14) Å^3. The values of R_p and R_{wp} were 5.189 and 8.518 %, respectively.

S6. Adsorption properties

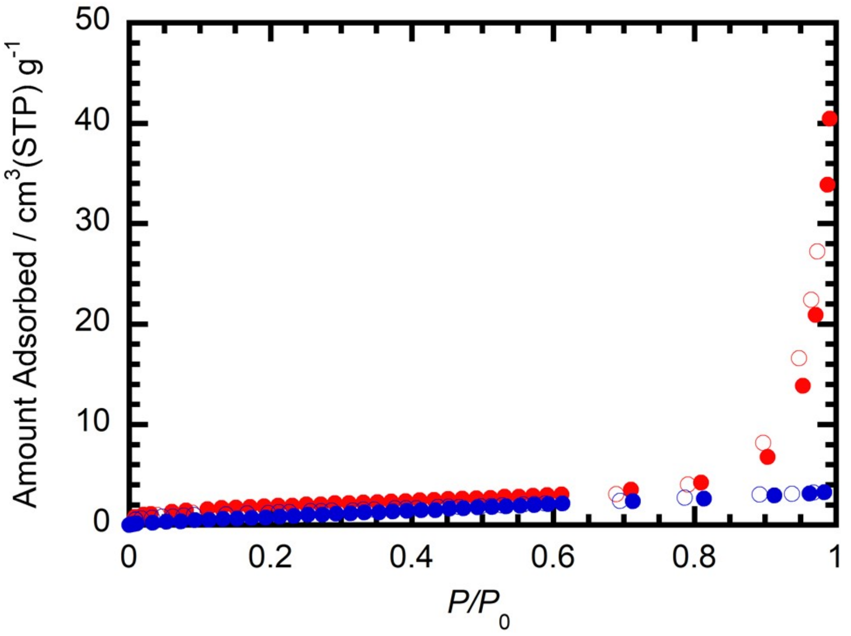

Fig. S8. N_2 (red) and CO_2 (blue) adsorption (filled symbols)/desorption (open symbols) isotherms at 77 and 195 K, respectively, for **1**.

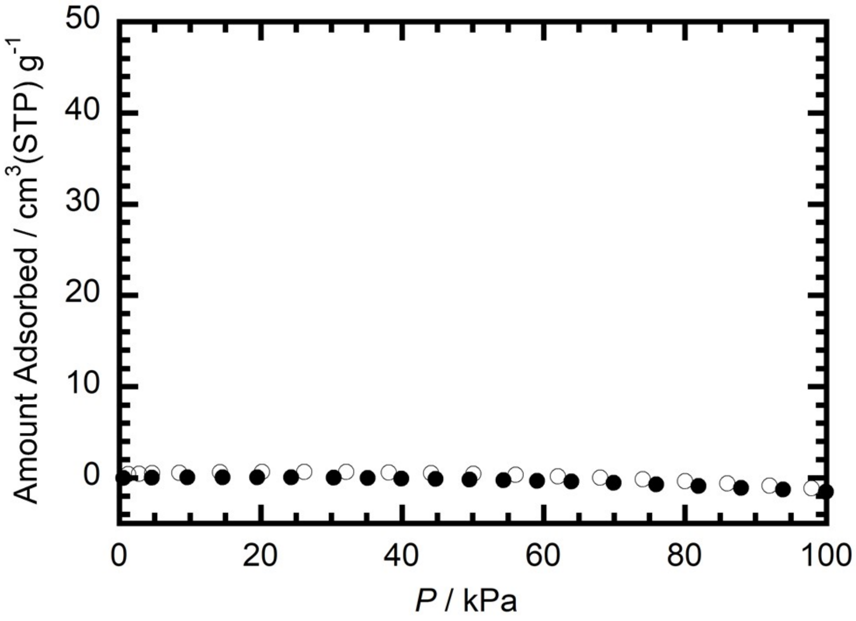

Fig. S9. CO_2 adsorption (filled symbols)/desorption (open symbols) isotherms at 298 K for **1**.

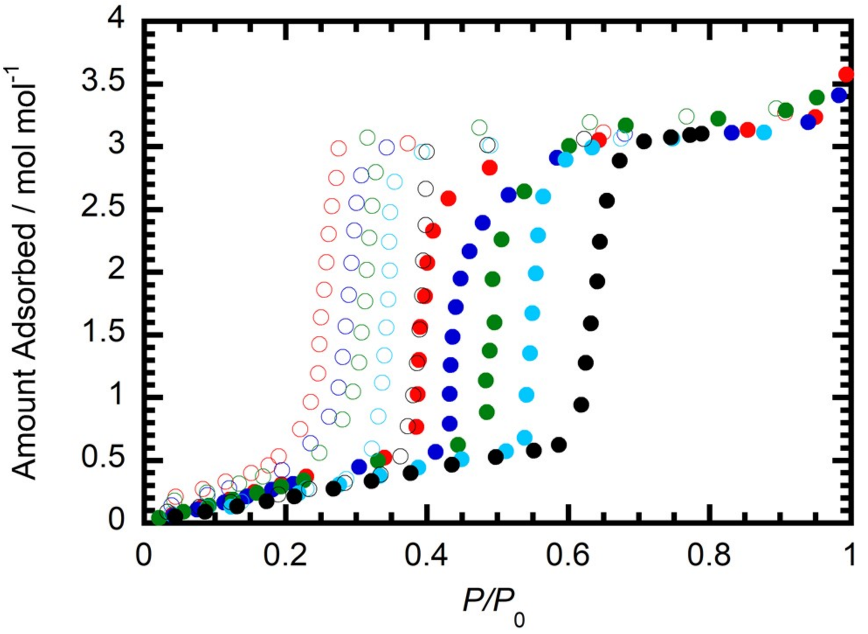

Fig. S10. H_2O adsorption (filled symbols)/desorption (open symbols) isotherms at 288 K (red), 298 K (blue), 308 K (green), 318 K (sky blue), and 328 K (black) for **1**.

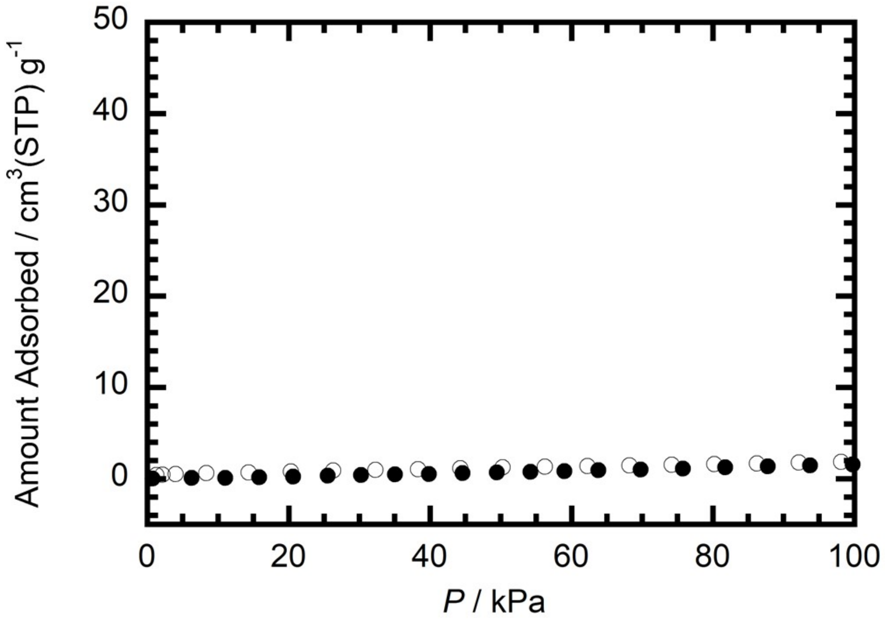

Fig. S11. CO_2 adsorption (filled symbols)/desorption (open symbols) isotherms at 298 K for $[Cu(CF_3SO_3)_2(bpp)_2]$.

S7. TG analysis under a moist gas flow

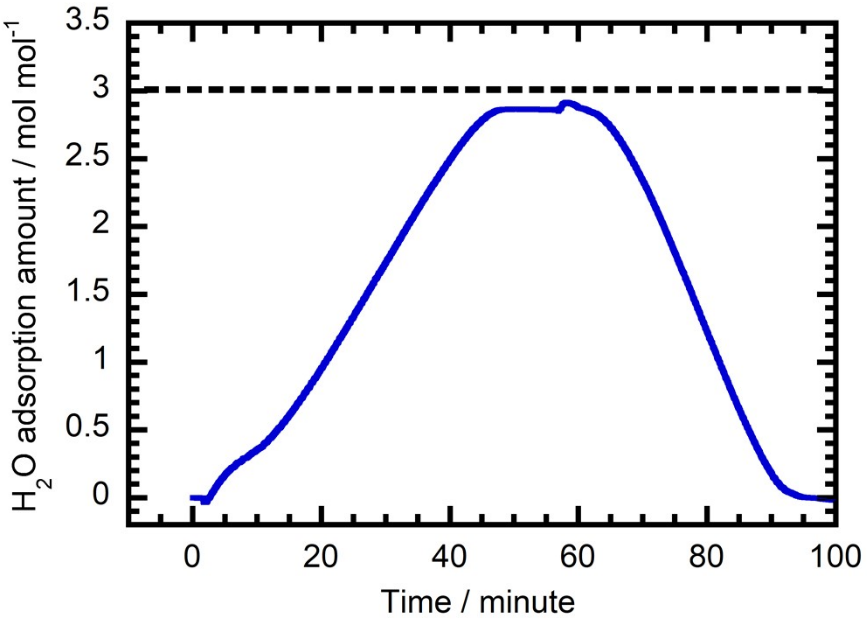

Fig. S12. Time dependence of the amount of H_2O adsorbed by **1** under humidified CO_2 (0-57 min) and dry CO_2 (57-100 min) at 298 K.

S8. IR spectroscopy under a moist gas flow

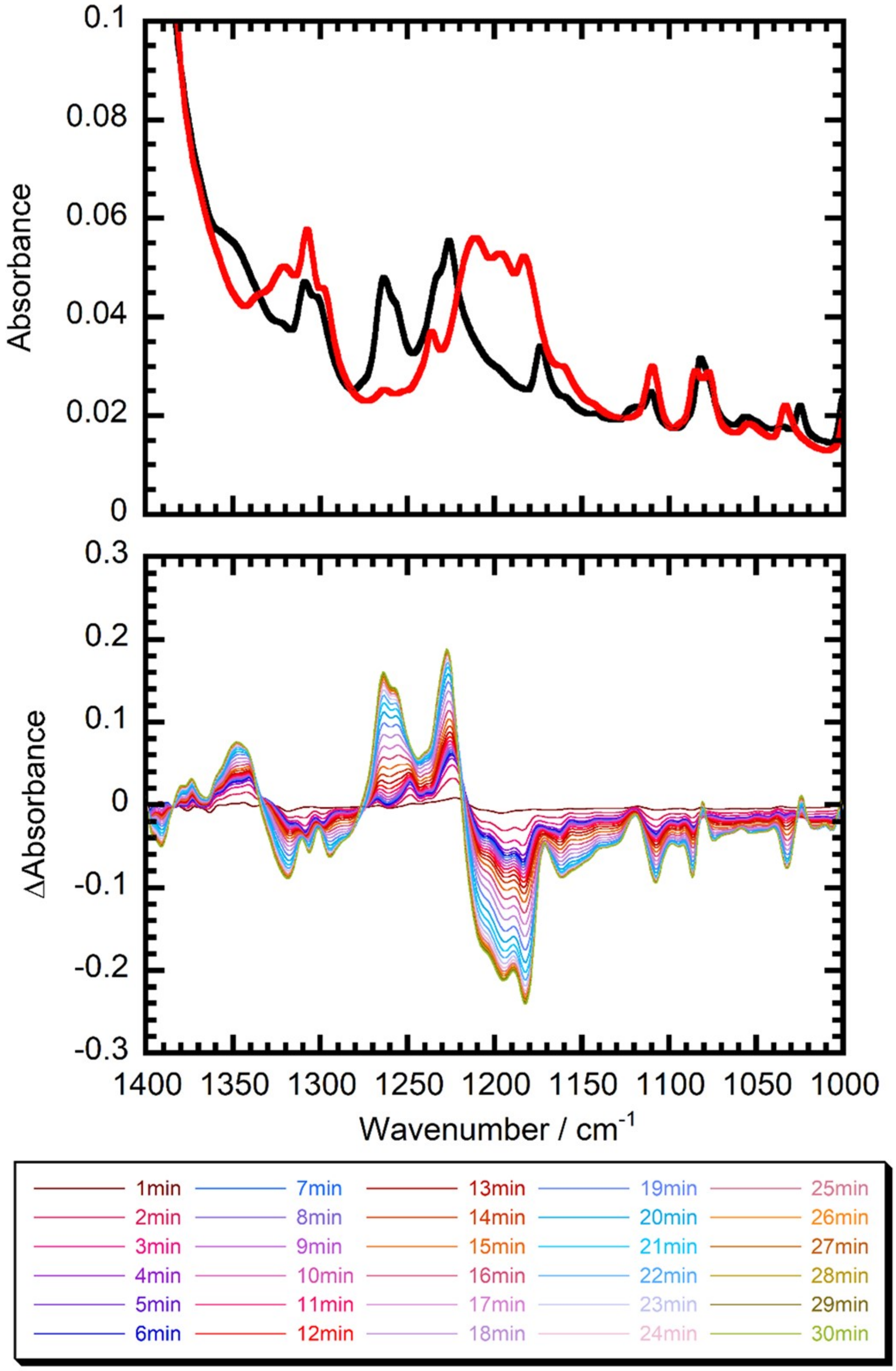

Fig. S13. ATR-IR spectra of **1** (red) and **1**·3H_2O (black) and difference IR spectra of **1** under wet CO_2 at 303 K; the displayed spectra were measured at 1 min intervals. At 0 min, the dry CO_2 gas was changed to wet CO_2 gas.

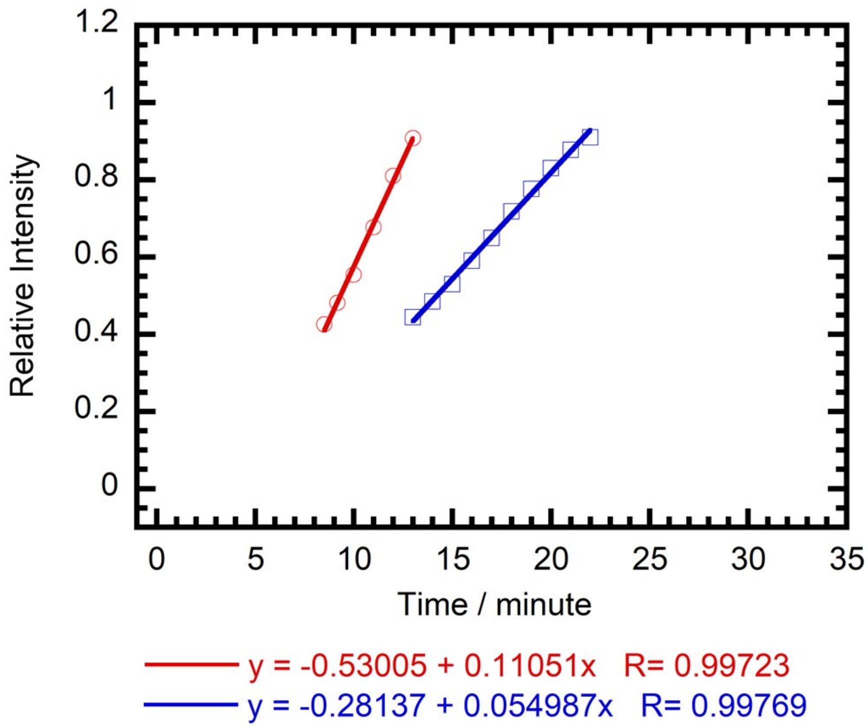

Fig. S14. Fitting of the data of time dependence of relative intensity for a peak at 1227 cm^{-1} under wet N_2 (red) and wet CO_2 (blue) at 303 K in **1**.

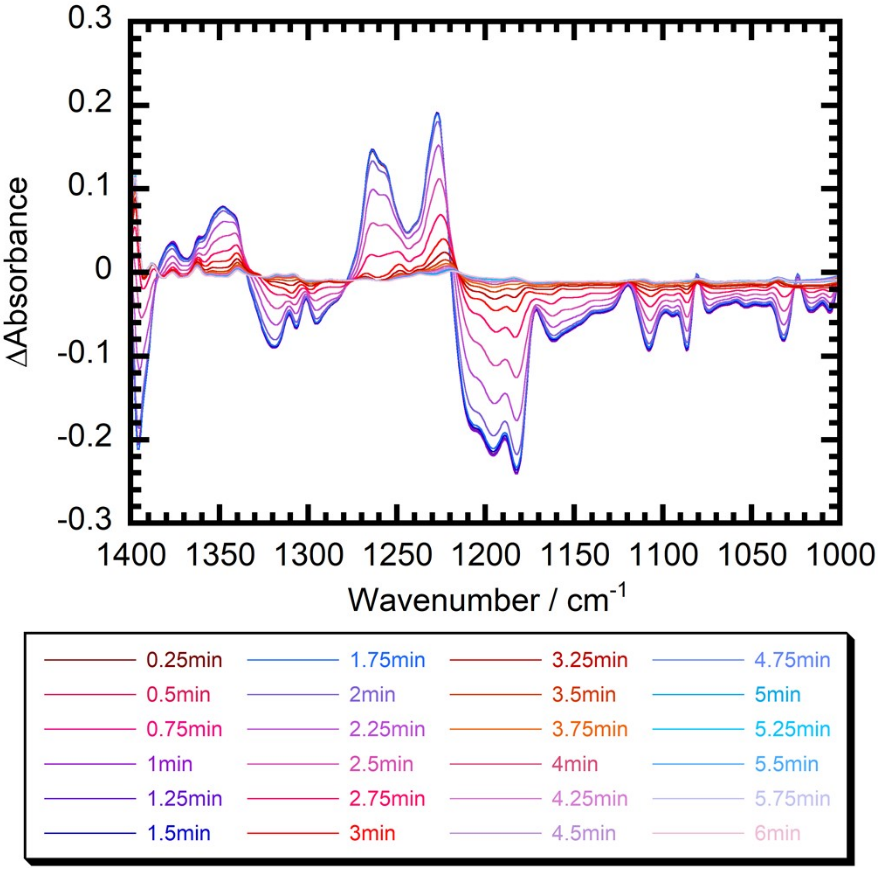

Fig. S15. Difference IR spectra of **1** under dry N_2 at 303 K; the displayed spectra were measured at 0.25 min intervals. At 0 min, the wet N_2 gas was changed to dry N_2 gas.

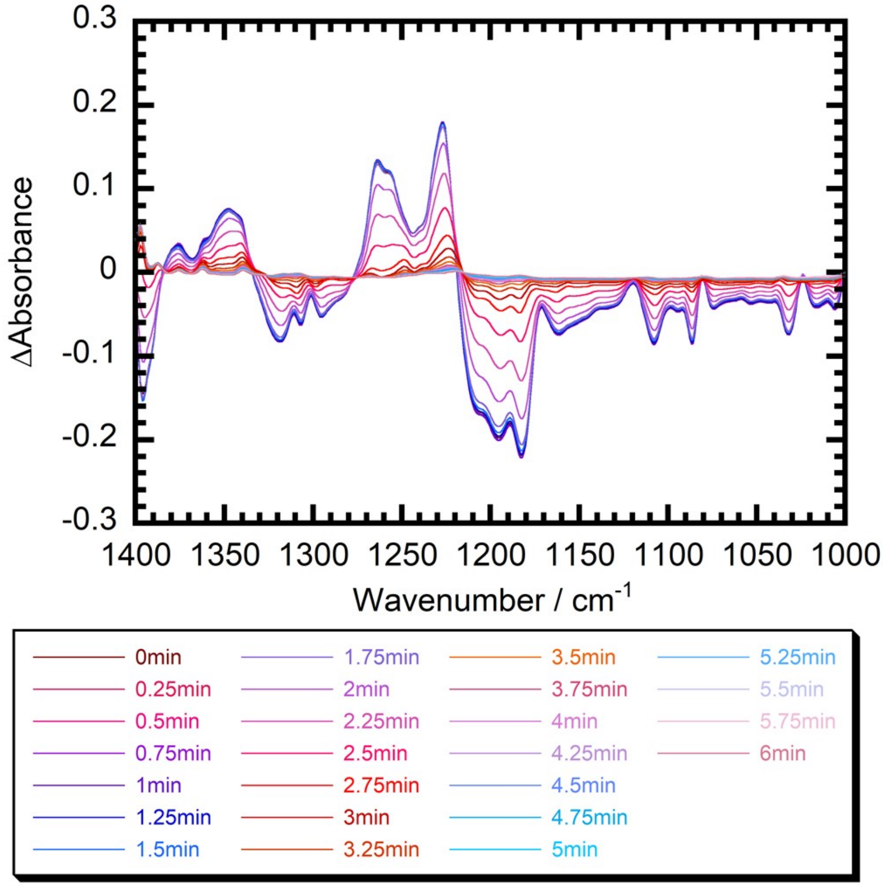

Fig. S16. Difference IR spectra of **1** under dry CO_2 at 303 K; the displayed spectra were measured at 0.25 min intervals. At 0 min, the wet CO_2 gas was changed to dry CO_2 gas.

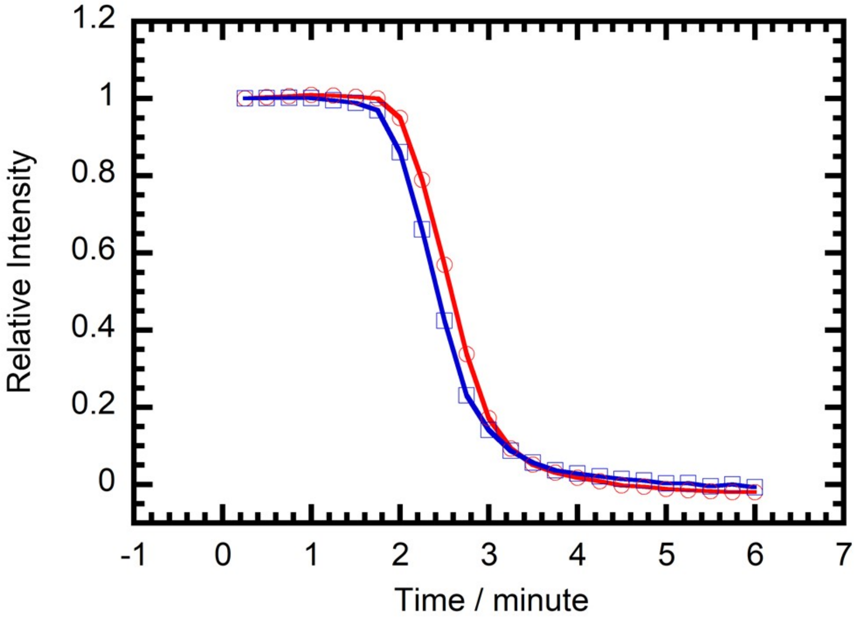

Fig. S17. Time dependence of relative intensity for a peak at 1227 cm^{-1} under dry N_2 (red) and dry CO_2 (blue) after humid N_2 and humid CO_2 flow at 303 K in **1**.

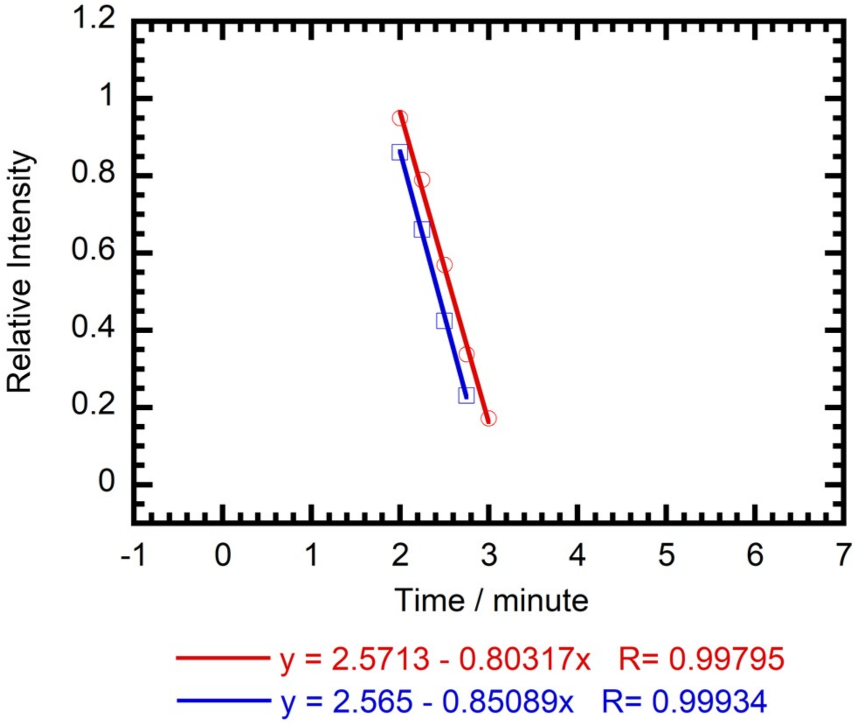

Fig. S18. Fitting of the data of time dependence of relative intensity for a peak at 1227 cm^{-1} under dry N_2 (red) and dry CO_2 (blue) after humid N_2 and humid CO_2 flow at 303 K in **1**.

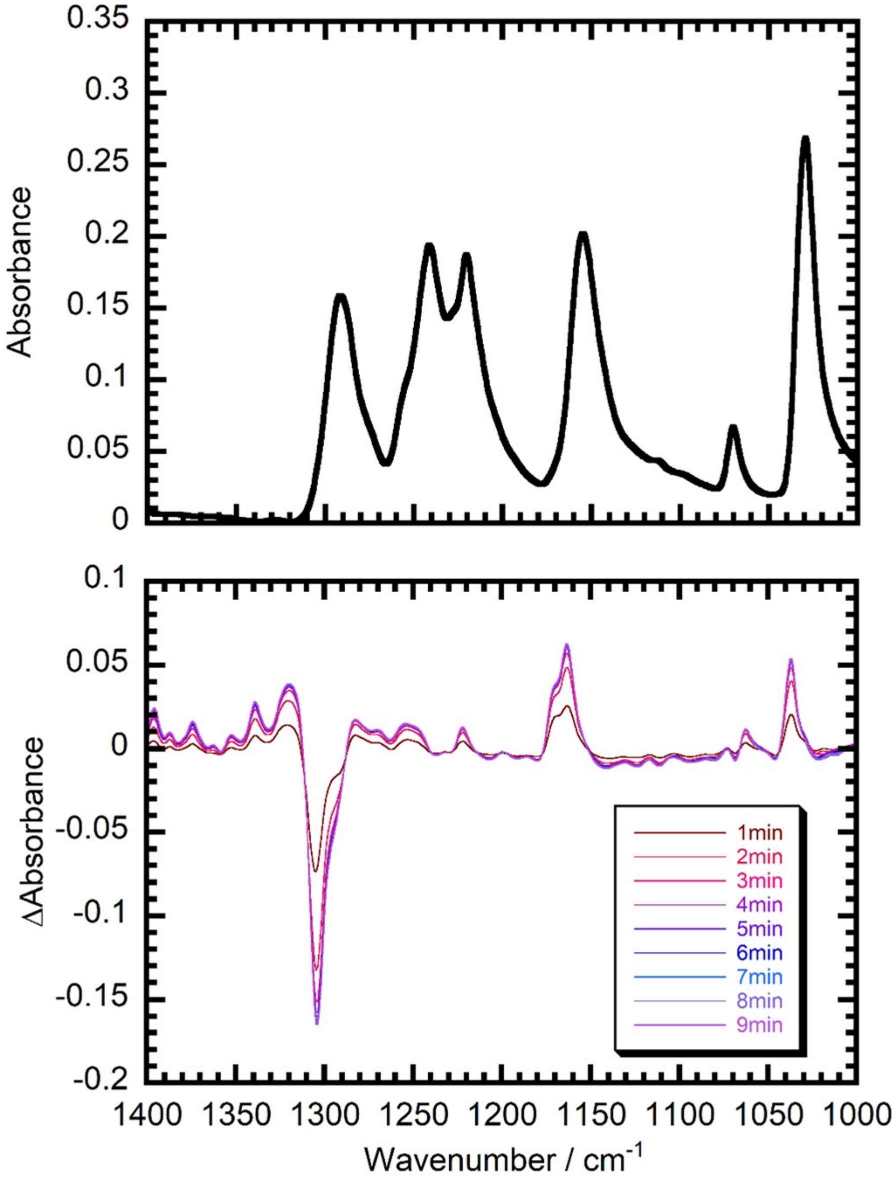

Fig. S19. ATR-IR spectra and difference IR spectra of $[Cu(CF_3SO_3)_2(bpp)_2]$ under wet N_2 at 303 K; the displayed spectra were measured at 1 min intervals. At 0 min, the dry N_2 gas was changed to wet N_2 gas.

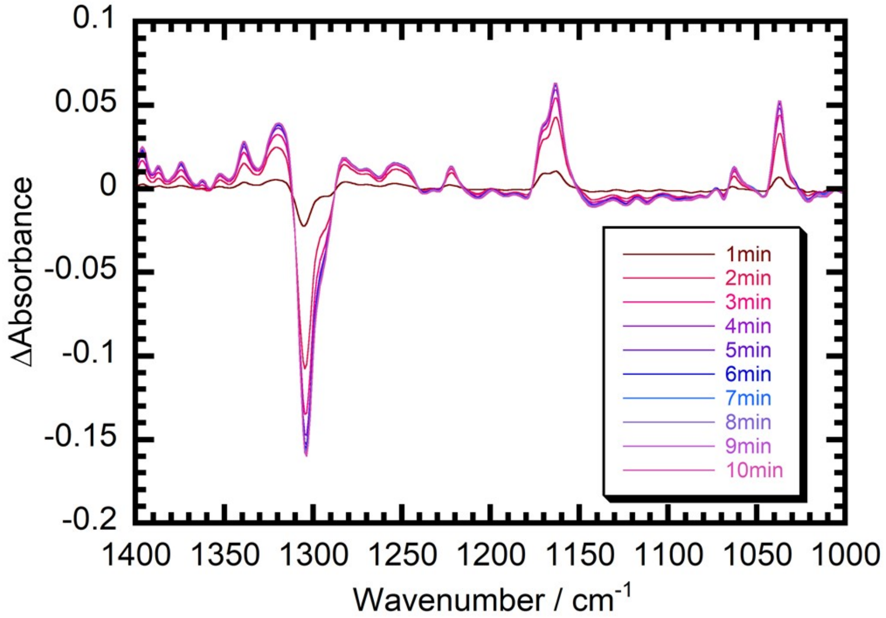

Fig. S20. Difference IR spectra of [Cu(CF_3SO_3)$_2$(bpp)$_2$] under wet CO_2 at 303 K; the displayed spectra were measured at 1 min intervals. At 0 min, the dry CO_2 gas was changed to wet CO_2 gas.

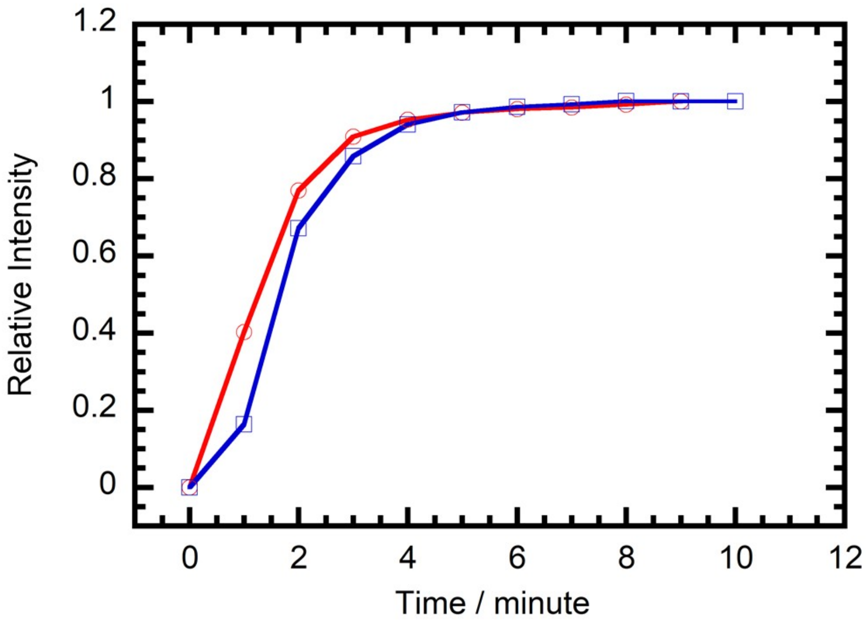

Fig. S21. Time dependence of relative intensity for a peak at 1163 cm^{-1} under humid N_2 (red) and humid CO_2 (blue) at 303 K in [Cu(CF_3SO_3)$_2$(bpp)$_2$].

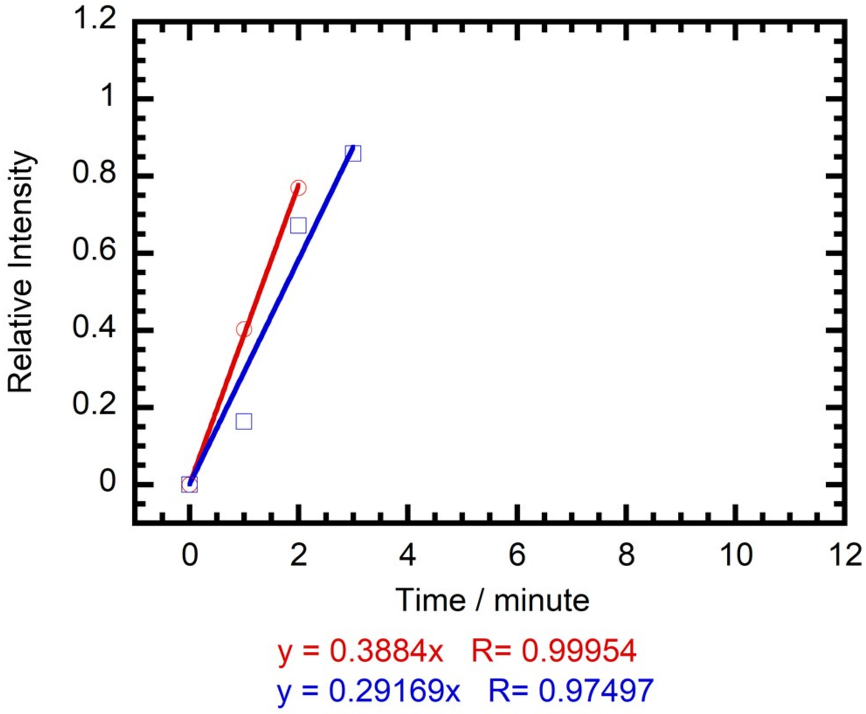

Fig. S22. Fitting of the data of time dependence of relative intensity for a peak at 1163 cm^{-1} under humid N_2 (red) and dry CO_2 (blue) at 303 K in [Cu(CF_3SO_3)$_2$(bpp)$_2$].

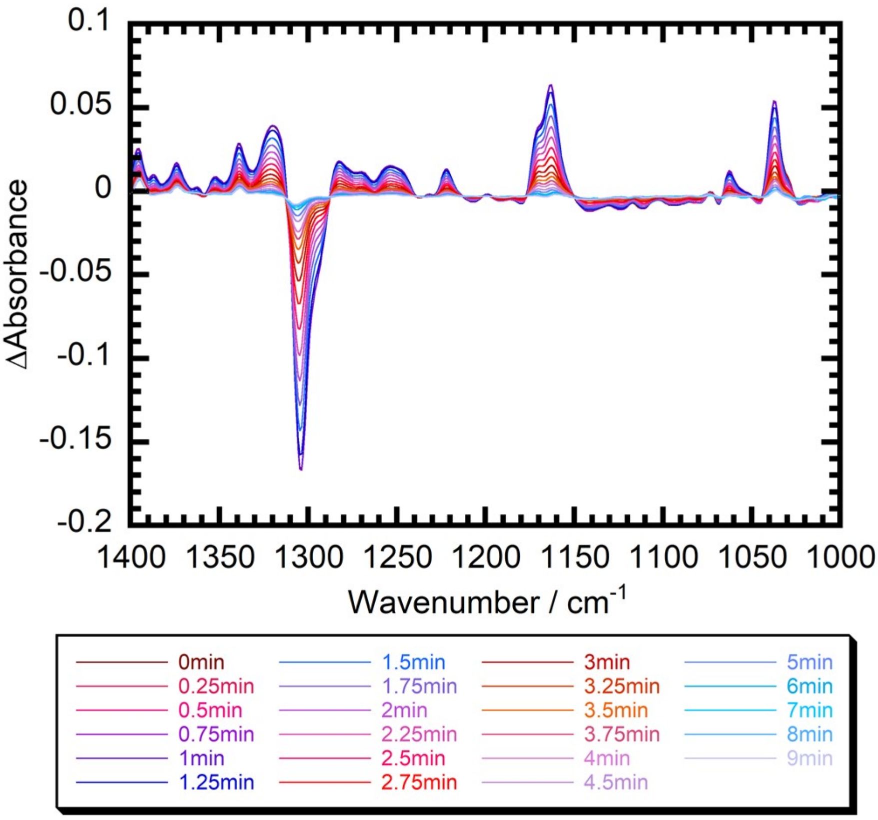

Fig. S23. Difference IR spectra of [$Cu(CF_3SO_3)_2(bpp)_2$] under dry N_2 at 303 K; the displayed spectra were measured at 0.25 min intervals between 0 and 4 min, 0.5 min intervals between 4 and 5 min, and 1 min intervals between 5 and 9 min. At 0 min, the wet N_2 gas was changed to dry N_2 gas.

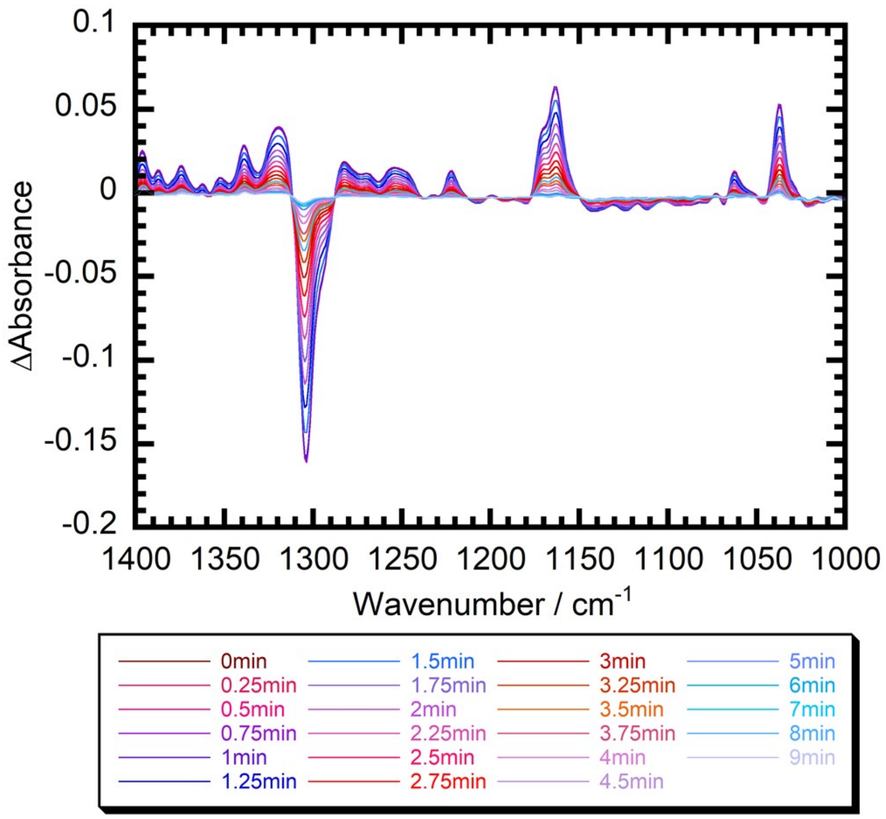

Fig. S24. Difference IR spectra of [Cu(CF_3SO_3)$_2$(bpp)$_2$] under dry CO_2 at 303 K; the displayed spectra were measured at 0.25 min intervals between 0 and 4 min, 0.5 min intervals between 4 and 5 min, and 1 min intervals between 5 and 9 min. At 0 min, the wet CO_2 gas was changed to dry CO_2 gas.

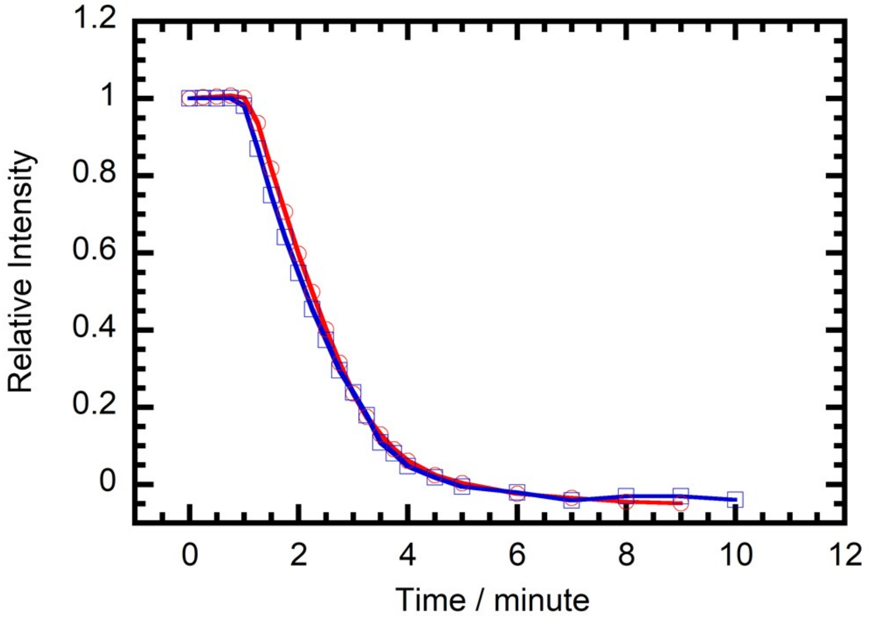

Fig. S25. Time dependence of relative intensity for a peak at 1163 cm^{-1} under dry N_2 (red) and dry CO_2 (blue) after humid N_2 and humid CO_2 flow at 303 K in $[Cu(CF_3SO_3)_2(bpp)_2]$.

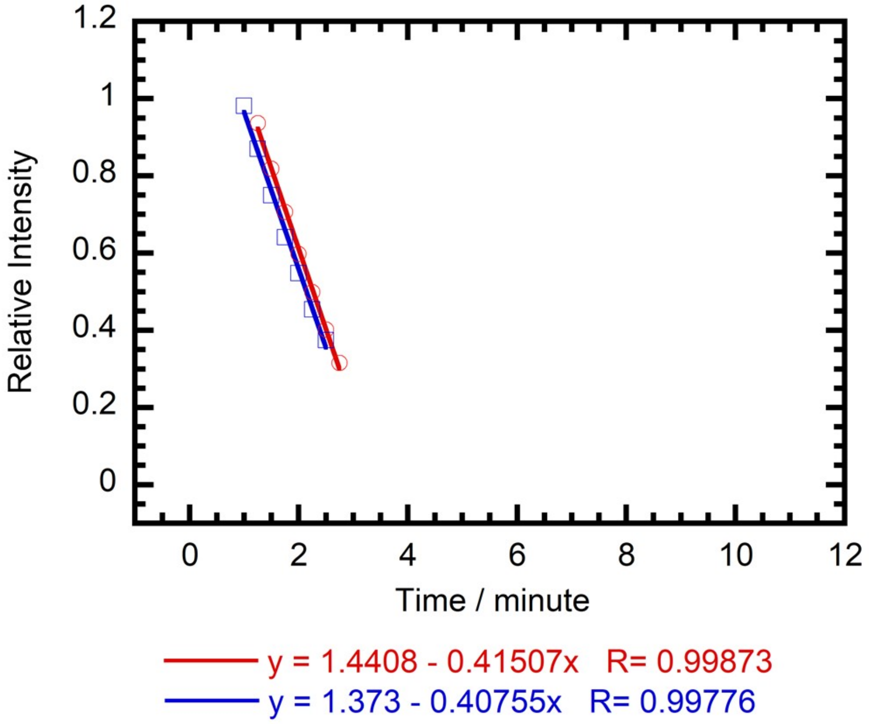

Fig. S26. Fitting of the data of time dependence of relative intensity for a peak at 1163 cm^{-1} under dry N_2 (red) and dry CO_2 (blue) after humid N_2 and humid CO_2 flow at 303 K in $[Cu(CF_3SO_3)_2(bpp)_2]$.

S9. References

S1 K. Fukuhara, S. Noro, K. Sugimoto, T. Akutagawa, K. Kubo, T. Nakamura, *Inorg. Chem.* 2013, **52**, 5630.

S2 A. Altomare, C. Cuocci, C. Giacovazzo, A. Moliterni, R. Rizzi, N. Corriero, A. Falcicchio, *J. Appl. Cryst.*, 2013, **46**, 1231.

S3 G. M. Sheldrick, *Acta Crystallogr.*, 2015, **A71**, 3.

S4 G. M. Sheldrick, *Acta Crystallogr.*, 2015, **C71**, 3.

S5 O. V. Dolomanov, L. J. Bourhis, R. J. Gildea, J. A. K. Howard, H. Puschmann, *J. Appl. Crystallogr.*, 2009, **42**, 339.
